# CDK1-CCNB1 creates a spindle checkpoint–permissive state by enabling MPS1 kinetochore localization

**DOI:** 10.1083/jcb.201808014

**Published:** 2019-01-23

**Authors:** Daniel Hayward, Tatiana Alfonso-Pérez, Michael J. Cundell, Michael Hopkins, James Holder, James Bancroft, Lukas H. Hutter, Bela Novak, Francis A. Barr, Ulrike Gruneberg

**Affiliations:** 1 Sir William Dunn School of Pathology, University of Oxford, South Parks Road, Oxford, England, UK; 2 Department of Biochemistry, University of Oxford, South Parks Road, Oxford, England, UK

## Abstract

Spindle checkpoint signaling is initiated by recruitment of the kinase MPS1 to unattached kinetochores during mitosis. We show that CDK1-CCNB1 and a counteracting phosphatase PP2A-B55 regulate the engagement of human MPS1 with unattached kinetochores by controlling the phosphorylation status of S281 in the kinetochore-binding domain. This regulation is essential for checkpoint signaling, since MPS1^S281A^ is not recruited to unattached kinetochores and fails to support the recruitment of other checkpoint proteins. Directly tethering MPS1^S281A^ to the kinetochore protein Mis12 bypasses this regulation and hence the requirement for S281 phosphorylation in checkpoint signaling. At the metaphase–anaphase transition, MPS1 S281 dephosphorylation is delayed because PP2A-B55 is negatively regulated by CDK1-CCNB1 and only becomes fully active once CCNB1 concentration falls below a characteristic threshold. This mechanism prolongs the checkpoint-responsive period when MPS1 can localize to kinetochores and enables a response to late-stage spindle defects. By acting together, CDK1-CCNB1 and PP2A-B55 thus create a spindle checkpoint–permissive state and ensure the fidelity of mitosis.

## Abstract (precis)

Hayward et al. show that CDK1-CCNB1 and PP2A-B55 limit spindle checkpoint signaling to a tightly defined window during mitosis. CDK1-CCNB1 promotes MPS1 localization to unattached kinetochores and thus creates a checkpoint-permissive state that is terminated by PP2A-B55 upon mitotic exit.

## Introduction

Error-free chromosome segregation is promoted by the coordinated actions of the cyclin B–dependent kinase (CDK1-CCNB), the ubiquitin E3 ligase anaphase promoting complex/cyclosome (APC/C), and the spindle checkpoint (Nurse, 1990; Nigg, 2001; Musacchio, 2015; Sivakumar and Gorbsky, 2015). Attachment of all the chromosomes within a cell to the mitotic spindle by their microtubule-binding kinetochores results in the APC/C-mediated ubiquitylation and proteasome-dependent destruction of securin, the inhibitor of chromosome segregation, and CCNB1, the inhibitor of mitotic exit (Novak et al., 2007; Sivakumar and Gorbsky, 2015). This results in chromosome segregation and a reduction in mitotic kinase activity enabling the onset of anaphase.

Mitotic exit is prevented by the spindle checkpoint pathway through the action of a conserved protein kinase Mono-Polar Spindles 1 (MPS1), which binds to kinetochores not attached to microtubules of the mitotic spindle (Abrieu et al., 2001; Stucke et al., 2002; Tighe et al., 2008; Liu and Winey, 2012; Musacchio, 2015). When localized to unattached kinetochores, MPS1 promotes the formation of a diffusible APC/C inhibitor, the mitotic checkpoint complex (MCC; Musacchio, 2015). This pool of MPS1 phosphorylates threonine residues in the MELT motifs in the kinetochore protein KNL1 (London et al., 2012; Shepperd et al., 2012; Yamagishi et al., 2012). MPS1-phosphorylated KNL1 is recognized by a complex of BUB3 and BUB1 (Primorac et al., 2013), which initiates a catalytic cycle involving MAD1, MAD2, BUBR1, and CDC20 that generates MCC (Faesen et al., 2017; Ji et al., 2017). MPS1 is lost from kinetochores attached to the mitotic spindle because of direct competition with microtubules for binding (Hiruma et al., 2015; Ji et al., 2015). These kinetochores therefore no longer catalyze MCC production due to a silencing process involving dephosphorylation of the MELT motifs, catalyzed directly or indirectly by BUBR1-associated PP2A-B56 (Espert et al., 2014; Nijenhuis et al., 2014). Once all kinetochores have attached to the mitotic spindle, APC/C inhibition is relieved due to cessation of MCC production and the disassembly of existing MCC (Sivakumar and Gorbsky, 2015). However, the checkpoint remains responsive to detachment of individual kinetochores from microtubules and can be reactivated for some time after the initial silencing event (Clute and Pines, 1999; Dick and Gerlich, 2013; Vázquez-Novelle et al., 2014). The precise length of this window is important: too short and the checkpoint may fail to detect attachment errors; too long and the APC/C would become reinhibited as sister chromatids separate and tension is lost, thus delaying cell division (Vázquez-Novelle et al., 2010; Kops, 2014). Mechanisms ensuring this precise timing is achieved are therefore thought to be crucial for genome stability.

The requirement for two other protein kinases, the mitotic kinase CDK1-CCNB and the centromeric Aurora B chromosomal passenger complex (CPC), has been proposed to limit MPS1-dependent spindle checkpoint signaling to mitosis (D’Angiolella et al., 2003; Vázquez-Novelle and Petronczki, 2010; Santaguida et al., 2011). MPS1 activity is directly regulated by CDK1 in *Xenopus laevis* egg extracts (Morin et al., 2012), and the drop in CDK1-CCNB activity in anaphase is required to limit the capacity of kinetochores to raise a checkpoint signal in human cells and mouse oocytes (Rattani et al., 2014; Vázquez-Novelle et al., 2014). Aurora B has a dual role in spindle checkpoint signaling by promoting both MPS1 recruitment to unattached kinetochores and the formation of unattached kinetochores through the prometaphase error-correction pathway (Lampson and Cheeseman, 2011; Foley and Kapoor, 2013). Both these functions involve HEC1, an outer kinetochore protein of the KMN complex which is directly involved in microtubule binding (Cheeseman et al., 2006; DeLuca et al., 2006). At unattached kinetochores or incorrect attachment geometries, situations that fail to generate tension across the centromere and kinetochores, centromeric Aurora B can reach and phosphorylate HEC1 (Cheeseman et al., 2006; DeLuca et al., 2006). Because Aurora B–phosphorylated HEC1 has a lower affinity for microtubules, this destabilizes incorrect attachments (Welburn et al., 2010). Furthermore, MPS1 directly interacts with Aurora B–phosphorylated HEC1, thus initiating a checkpoint signal (Saurin et al., 2011; Nijenhuis et al., 2013; Zhu et al., 2013). At amphitelic tension-generating attachments, centromeric Aurora B is physically separated from the outer kinetochore. A kinetochore pool of PP2A-B56, initially recruited by MPS1-dependent checkpoint signaling through BUBR1, then counteracts Aurora B and thus stabilizes these attachments (Foley et al., 2011; Suijkerbuijk et al., 2012; Kruse et al., 2013). In this way, Aurora B and PP2A-B56 support a cycle of error correction and checkpoint signaling until all attachment errors have been resolved. Because of its role in promoting Aurora B localization to centromeres (Tsukahara et al., 2010; Zhou et al., 2014), CDK1-CCNB thus acts upstream of the error-correction and checkpoint signaling pathways.

At the onset of anaphase, Aurora B is transported away from the centromere by a CDK1-inhibited process involving the mitotic kinesin MKLP2 (Gruneberg et al., 2004; Hümmer and Mayer, 2009; Kitagawa et al., 2014). This will inevitably compromise both the spindle checkpoint and error-correction pathways. However, centromeric retention of Aurora B in anaphase in MKLP2-depleted cells does not enable spindle checkpoint signaling in anaphase or delay mitotic exit (Vázquez-Novelle and Petronczki, 2010), suggesting there must be an additional limiting factor. An obvious candidate is CDK1-CCNB, which, as we have explained, is required for the activities of both MPS1 and Aurora B. We therefore propose that CDK1-CCNB should be considered an upstream regulatory component required to create a spindle checkpoint–permissive state.

A crucial aspect of this model for the creation of a checkpoint-permissive state is the requirement for a CDK1-inhibited counteracting phosphatase acting on CDK1 targets directly involved in generating a checkpoint signal at unattached kinetochores (He et al., 2011). As already discussed, both MPS1 and the Aurora B CPC are candidate targets for CDK1-CCNB. However, the CDK1-inhibited counteracting phosphatase required to revoke the checkpoint-permissive state has not been defined. Two known enzymes could fulfil this role, PP1 and PP2A-B55 (Wurzenberger and Gerlich, 2011). PP1 is directly inhibited by CDK1 phosphorylation of a conserved C-terminal motif (Dohadwala et al., 1994; Kwon et al., 1997), whereas PP2A-B55 is inhibited by phosphorylated members of the ENSA/ARRP19 family, which compete for substrate binding (Gharbi-Ayachi et al., 2010; Mochida et al., 2010; Williams et al., 2014). These B55 inhibitors are phosphorylated by the kinase Greatwall/MASTL, which is activated by CDK1 phosphorylation, thus reducing PP2A-B55 activity in mitosis (Yu et al., 2006; Vigneron et al., 2011; Blake-Hodek et al., 2012). Removal of either ENSA/ARPP19 or Greatwall/MASTL therefore leads to constitutive maximal PP2A-B55 activity and dysregulated mitotic exit (Burgess et al., 2010; Cundell et al., 2013). However, these defects are not detected by the spindle checkpoint, and the cells undergo precocious cytokinesis leading to a catastrophic failure of cell division (Burgess et al., 2010; Manchado et al., 2010; Voets and Wolthuis, 2010; Cundell et al., 2013; Diril et al., 2016).

In this study, we investigate the roles of CDK1-CCNB1, PP1, and PP2A-B55 in regulation of MPS1 and Aurora B localization and checkpoint signaling. We propose that CDK1-CCNB1 and PP2A-B55 constitute a regulatory system for MPS1 and Aurora B and thus control the period in which the mitotic spindle is surveyed for defects by the spindle checkpoint.

## Results

### PP2A-B55 counteracts CDK-dependent checkpoint signaling

**Figure 1.**
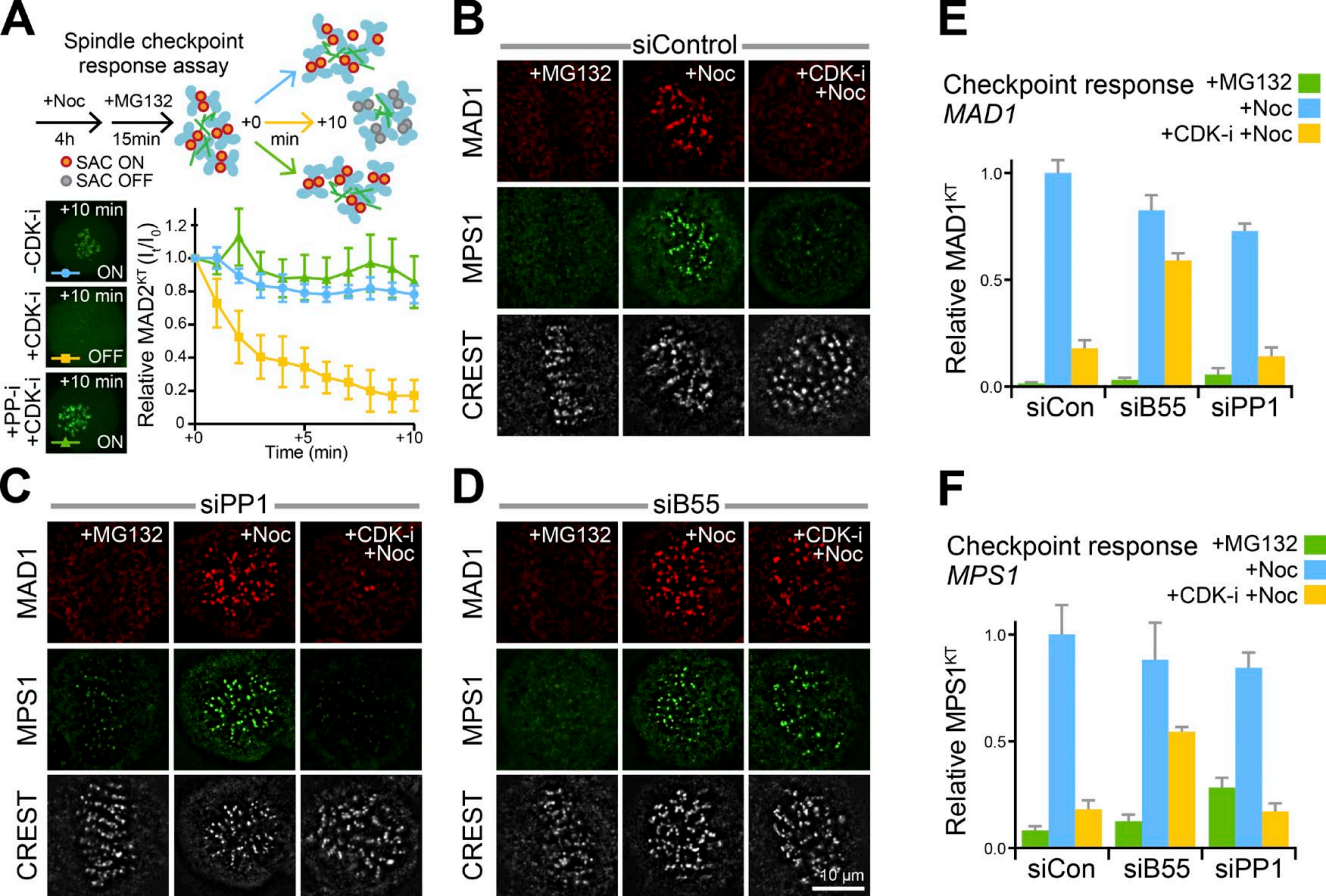
**PP2A-B55 counteracts CDK1-dependent MPS1 localization to kinetochores and checkpoint signaling. (A)** Spindle checkpoint response assays are explained in the schematic. HeLa GFP-MAD2 cells arrested in mitosis with 0.3 µM nocodazole for 4 h were treated with 20 µM MG132 for 15 min to prevent mitotic exit. Images were taken every minute after treatment with DMSO (−CDK-i control), 5 µM flavopiridol alone (+CDK-i), or 25 nM calyculin A for 1 min before 5 µM flavopridol addition (PP-i +CDK-i). Representative images at +10 min are shown. Mean GFP-MAD2 intensity at kinetochores as a function of time (I_t_) is plotted relative to the starting signal (I_0_); error bars indicate the SEM (*n* ≥ 5 independent conditions). **(B–D)** Checkpoint signaling and endogenous MPS1 localization were followed in control (siControl; B), PP1αγ-depleted (siPP1; C), and PP2A-B55–depleted (siB55) HeLa MPS1-GFP cells (D). Cells were arrested for 2.5 h with 20 µM MG132 and then either fixed immediately (+MG132), treated with 3 µM nocodazole for 5 min (+Noc), or 5 µM flavopiridol for 1 min, followed by addition of 3 µM nocodazole for 5 min (+CDK-i +Noc). MAD1 and kinetochores (CREST) were detected using antibodies, and MPS1 was detected using GFP fluorescence. **(E and F)** Kinetochore-associated MAD1 (E) and MPS1-GFP normalized to the mean of the nocodazole-treated control (F) are plotted; error bars indicate the SEM (15 cells and 15 kinetochores measured per cell for three independent experiments).

To investigate the requirements for CDK activity and a counteracting phosphatase in spindle checkpoint signaling, cells expressing the checkpoint marker GFP-MAD2 were treated with a combination of the microtubule poison nocodazole and the proteasome inhibitor MG132 (Fig. 1 A and Video 1). As expected, these cells were arrested in mitosis and the checkpoint, judged by the presence of MAD2-positive kinetochores, remained active (Fig. 1 A, −CDK-i). Following CDK-inhibition, this MAD2 checkpoint signal was rapidly lost from kinetochores (Fig. 1 A, +CDK-i). Importantly, loss of MAD2 from kinetochores was prevented by the addition of a broad spectrum PP1 and PP2A inhibitor, calyculin A (Fig. 1 A, +PP-i +CDK-i). These results show that not only is CDK activity required to create a spindle checkpoint–permissive state during mitosis, a PP1 or PP2A-family phosphatase is necessary to revoke this.

To identify the specific phosphatase, endogenous MPS1 was followed in checkpoint-silenced (metaphase) HeLa MPS1-GFP cells (Fig. S1 A), arrested downstream of the Aurora B and PP2A-B56–dependent error-correction process by proteasome inhibition. These cells were tested for the ability to recruit MPS1 to kinetochores and reactivate the checkpoint under conditions where CDK activity was maintained or lost. Under control conditions, addition of nocodazole triggered a rapid CDK-dependent recruitment of MPS1, MAD1, BUB1, and BUBR1 to kinetochores (Fig. 1 B and Fig. S1 B), demonstrating checkpoint reactivation had occurred. This behavior was not altered by depletion of PP1 (Fig. 1 C) or by inhibition of PP1 using the selective inhibitor tautomycetin (Fig. S1, D–G; +PP1-i; Choy et al., 2017). To confirm PP1 inhibition, histone threonine 3 phosphorylation by Haspin was used (Qian et al., 2011; De Antoni et al., 2012; Espert et al., 2014). In both cases of PP1 depletion or inhibition, reduction of phosphatase activity was confirmed by retention of histone H3 threonine 3 phosphorylation following Haspin inhibition (Fig. S1, H and I). In contrast to PP1, depletion of PP2A-B55 enabled the recruitment of MPS1, MAD1, BUB1, and BUBR1 to kinetochores and hence reactivation of checkpoint signaling in the absence of ongoing CDK activity (Fig. 1 D and Fig. S1 B). Quantification of the kinetochore pools of MAD1 and MPS1 revealed that these were penetrant effects (Fig. 1, E and F), and blotting confirmed depletion of the relevant PP1 and PP2A-B55 subunits (Fig. S1 C). These results were confirmed in untransformed, near-diploid telomerase immortalized human retinal pigment epithelial cells (hTERT-RPE1 cells), demonstrating that our findings are not obviously dependent on the ploidy or transformation status of the cells used (Fig. S1, F and G). Finally, kinetochore localization of the MPS1 receptor HEC1 was unaffected under these conditions (Fig. S1 J), supporting the view that altered checkpoint signaling, rather than disassembly of the kinetochore structure, explains these observations (Gascoigne and Cheeseman, 2013; Nijenhuis et al., 2013; Zhu et al., 2013; Hiruma et al., 2015; Ji et al., 2015). Collectively, these results show that PP2A-B55 counteracts CDK1 in MPS1 localization and may thus have an important role in spindle checkpoint signaling.

### Spindle checkpoint arrest is extended in the absence of PP2A-B55

**Figure 2.**
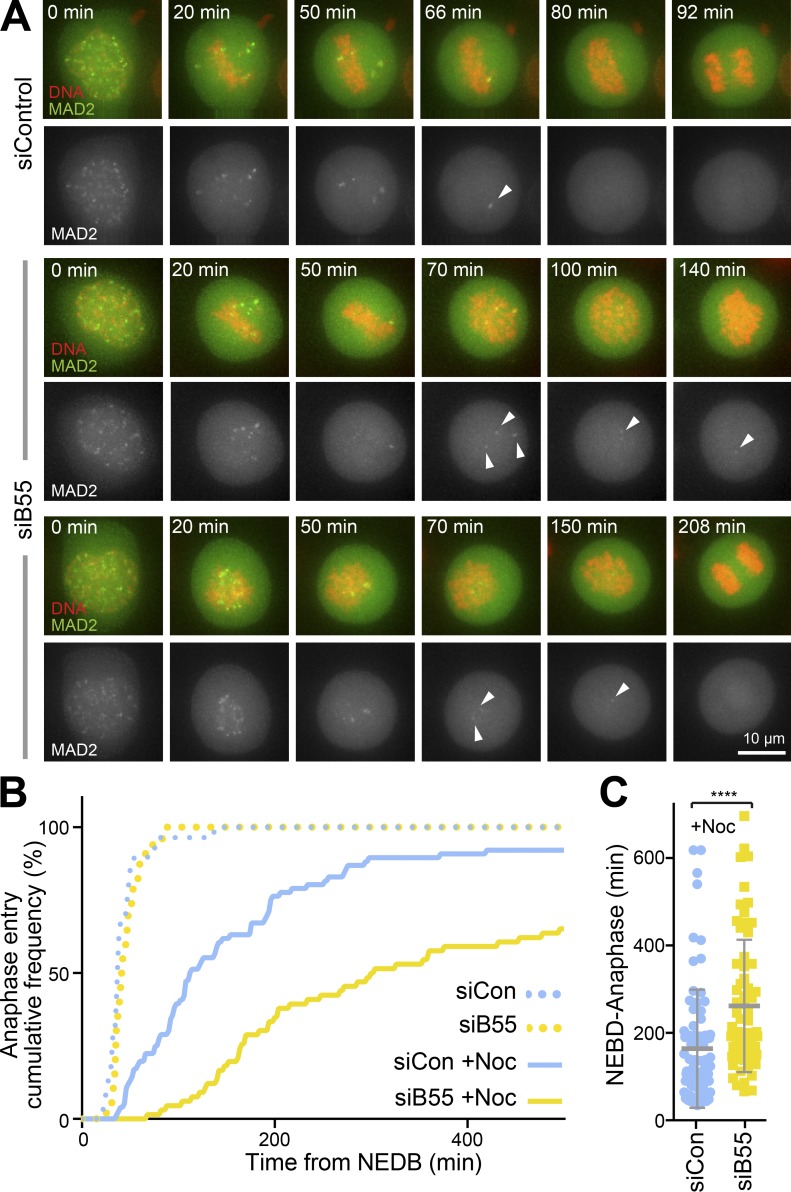
**Extended checkpoint responsiveness in the absence of PP2A-B55. (A)** Control (siControl) and PP2A-B55–depleted (siB55) Hela GFP-MAD2 cells, left untreated or exposed to 8.25 nM nocodazole, were imaged every 2 min passing through mitosis. Images show the behavior of MAD2 in the presence of 8.25 nM nocodazole with two examples of the siB55 condition. **(B)** Cumulative mitotic exit frequency was measured in the absence (siCon; *n* = 28) or presence of 8.25 nM nocodazole (siCon +Noc; *n* = 76) and following PP2A-B55 depletion in the absence (siB55; *n* = 33) or presence of 8.25 nM nocodazole (siB55 +Noc; *n* = 66). **(C)** NEBD-anaphase duration in 8.25 nM nocodazole is plotted; each point represents an individual cell with mean NEBD-anaphase time and SD shown as bars. ****, P < 0.0001 (Student’s *t* test).

To better understand the role of PP2A-B55 in checkpoint signaling, HeLa cells stably expressing GFP-MAD2 were imaged using time-lapse microscopy in the presence or absence of a low 8.25 nM dose of nocodazole, designed to mimic physiological disturbances of microtubule behavior as caused by, e.g., small temperature changes. In the presence of a low dose of nocodazole, cells lacking PP2A-B55 progressed into anaphase with significantly lower frequency than control cells and displayed persistent reappearance of GFP-MAD2 signals at single kinetochores (Fig. 2 A, arrowheads; and Fig. 2 B, solid lines). Under these conditions, the half-time for progression through mitosis from nuclear envelope breakdown (NEBD) to anaphase onset was increased from 175 to 250 min in B55-depleted cells (Fig. 2 C). In the absence of nocodazole, control and B55-depleted cells proceeded into anaphase with identical kinetics (Fig. 2 B, dotted lines), confirming previous results demonstrating that PP2A-B55 is not required for the metaphase to anaphase transition under unperturbed conditions (Cundell et al., 2013). Our new data indicate that in the absence of PP2A-B55 the metaphase-to-anaphase transition becomes unreliable when microtubule–kinetochore attachment is subject to mild perturbation and that, accordingly, PP2A-B55 has an important, hitherto unrecognized role in spindle checkpoint signaling.

The data presented thus far show that CDK1 and the CDK1-inhibited PP2A-B55 phosphatase regulate the checkpoint-permissive state, consistent with the central feature of our previously published checkpoint-signaling model (He et al., 2011). A further prediction of this model is that both activities converge on a common target responding to the presence of unattached kinetochores, and this was investigated next.

### MPS1 is a target of the B55-regulated exit pathway

**Figure 3.**
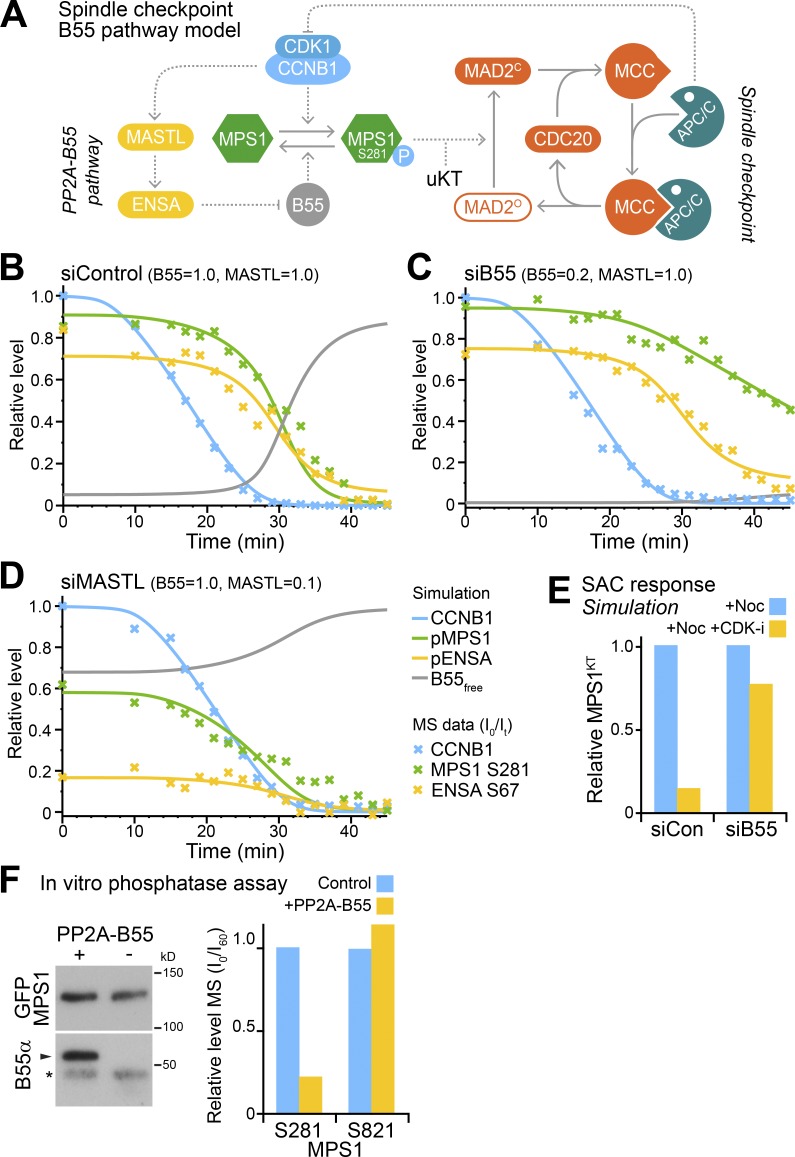
**MPS1 is a target of the B55-regulated exit pathway. (A)** Unified wiring diagram for the spindle checkpoint and PP2A-B55 pathway. **(B–D)** Simulation of the system described in the wiring diagram was performed for control (siControl; B), 80% PP2A-B55–depleted (siB55 0.2; C), and 90% MASTL-depleted (siMASTL 0.1; D) conditions. Experimental data points obtained by mass spectrometry are marked by an X, while simulated output is shown in solid lines. **(E)** Simulation of checkpoint response under conditions used for the checkpoint response assays in Fig. 1 (B and D). **(F)** Mitotically phosphorylated MPS1-GFP purified from 20 mg of cell lysate was incubated with purified PP2A-B55 or buffer control, and dephosphorylation was analyzed by mass spectrometry. The intensities of the phospho-peptides covering S281 and S821 were plotted.

We can now extend our previously established model for checkpoint signaling (He et al., 2011) by defining the CDK-inhibited counteracting phosphatase as PP2A-B55 (Fig. 3 A). This revised model also incorporates the MASTL and ENSA components of the PP2A-B55–regulatory pathway. To identify candidate spindle checkpoint targets regulated by both CDK1 and PP2A-B55, cellular protein phosphorylation levels were measured to high temporal resolution using mass spectrometry in synchronized cells exiting mitosis under checkpoint regulated conditions (Fig. S2 A). For this purpose, three conditions were compared: control (siControl), reduced PP2A-B55 activity (siB55), or increased unregulated PP2A-B55 activity (siMASTL). Initial blot analysis showed that cells exited mitosis under all three conditions and confirmed depletion of the relevant proteins (Fig. S2, B and C). This result is important since our model predicts that the PP2A-B55 pathway, although important for enabling a spindle checkpoint signaling at unattached kinetochores, is not required for checkpoint silencing in metaphase. PP2A-B55–regulated phosphorylation sites display characteristic dephosphorylation kinetics during passage from metaphase to anaphase (Fig. S2 A, right; Cundell et al., 2016). Intriguingly, the dephosphorylation kinetics of MPS1 phospho-Ser281 (pS281), a reported CDK1 site in the N terminus of the spindle checkpoint master regulator MPS1 (Dou et al., 2011; Morin et al., 2012) matched this expected kinetic profile for a bona fide PP2A-B55 target. MPS1 S281 showed delayed sigmoidal dephosphorylation kinetics in control cells exiting mitosis (Fig. 3 B, marked data points). This was further delayed when PP2A-B55 was depleted (Fig. 3 C) and accelerated when MASTL was depleted (Fig. 3 D). Furthermore, the steady-state level of MPS1 S281 phosphorylation in the starting metaphase conditions was reduced in MASTL-depleted cells compared with the control (Fig. 3 D and Fig. S2 E). MPS1 phosphorylation sites at S393, S436, and S821, identified by mass spectrometry, also turned over during mitotic exit (Fig. S2 F), but of the candidate CDK sites, only S281 showed clear hallmarks of PP2A-B55 regulation.

These observations are consistent with the proposal that regulated PP2A-B55 activity determines both the timing of MPS1 S281 dephosphorylation and steady-state level of phosphorylation required to sustain a checkpoint-permissive state. To further support this view, the phosphorylation state of bulk MPS1 was simulated by the unified checkpoint model. The activities of CDK1-CCNB1 and the inhibitor of PP2A-B55 under the different conditions and depletion efficiencies were established by blotting and mass spectrometry of CCNB1 and ENSA phosphorylated at S67 (Fig. S2, D and E; and Table S1). These constraints were used to simulate the system described by the wiring diagram in Fig. 3 A under siControl, siB55, and siMASTL conditions. This approach provided estimates for the kinetic parameters used in the subsequent modeling. Dephosphorylation of MPS1 S281 (pMPS1), destruction of CCNB1, dephosphorylation of the PP2A-B55 inhibitor ENSA (pENSA), and activation of PP2A-B55 were accurately captured by the model (Fig. 3, B–D; solid lines). The model has also been used to calculate MPS1 dynamics at kinetochores in control and PP2A-B55–depleted conditions, following checkpoint reactivation with nocodazole. Again, the simulation of MPS1 levels at kinetochores (Fig. 3 E) was well matched to experimental data (Fig. 1, B, D, and F).

To confirm that MPS1 pS281 is a direct PP2A-B55 substrate, MPS1-GFP isolated from mitotic cells was incubated with purified PP2A-B55 or a buffer control in vitro*,* and the phosphorylation status of S281 assessed by mass spectrometry. Phosphorylation on MPS1 S281, but not S821, was markedly reduced after incubation with PP2A-B55, corroborating our in vivo results that S281 dephosphorylation is dependent on PP2A-B55 (Fig. 3 F). In summary, our data show that MPS1 S281 is a target for both CDK1 and PP2A-B55.

### CDK1 phosphorylation promotes MPS1 localization to kinetochores

**Figure 4.**
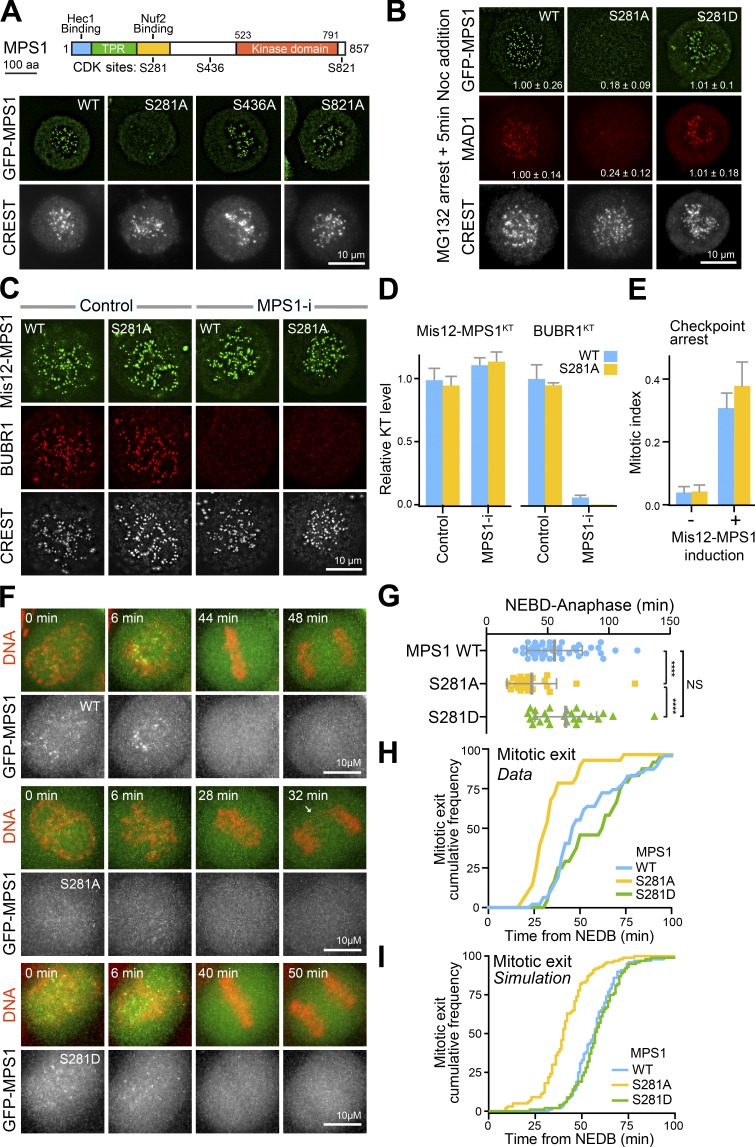
**Phosphorylation of MPS1 at S281 regulates its kinetochore localization and checkpoint signaling. (A)** Domain organization, functional binding sites, and mapped CDK sites of *Homo sapiens* MPS1. HeLa Flp-In/TREx GFP-MPS1^WT^, GFP-MPS1^S281A^, MPS1S^436A^, or MPS1^S821A^ cells were depleted of endogenous MPS1. GFP-MPS1 transgenes were induced for a total of 54 h, and then the cells were treated with 0.1 µM Taxol for 2 h. To prevent exit from mitosis, 20 µM MG132 was added for the last 30 min. Cells were stained with antibodies for kinetochores (CREST). **(B)** HeLa Flp-In/TREx GFP-MPS1^WT^, GFP-MPS1^S281A^, or GFP-MPS1^S281D^ cells were depleted of endogenous MPS1. GFP-MPS1 transgenes were induced, and after 24 h, the cells were arrested for 2.5 h with 20 µM MG132, treated with 3 µM nocodazole for 5 min, and then stained with antibodies for MAD1 and kinetochores (CREST). Kinetochore-associated GFP-MPS1 or MAD1 normalized to the mean of the MPS1^WT^ condition are indicated as the mean ± SEM (*n* ≥ 25 cells and 15 kinetochores measured per cell in four independent experiments). **(C)** HeLa Flp-In/TREx GFP-Mis12-MPS1^WT^ or GFP-Mis12-MPS1^S281A^ cells were depleted of endogenous MPS1. GFP-Mis12-MPS1 transgenes were induced (+) for 24 h and the cells treated with 0.1 µM Taxol for 2 h and either DMSO (Control) or 2 µM AZ3146 (MPS1-i) for 10 min. Cells were stained with antibodies for BUBR1 and kinetochores (CREST). Mis12-MPS1 was visualized by GFP fluorescence. **(D)** The bar graphs show the mean levels of Mis12-MPS1^WT^, Mis12-MPS1^S281A^, and BUBR1 at kinetochores in both control and MPS1-inhibited cells (MPS1-i). Bars indicate the SEM (15 kinetochores per cell and 12 cells per condition in three independent experiments). **(E)** GFP-Mis12-MPS1 transgenes were induced for 48 h in HeLa Flp-In/TREx GFP-Mis12-MPS1^WT^ or GFP-Mis12-MPS1^S281A^ cells depleted of endogenous MPS1, and the mitotic index was scored. The graph shows the mean mitotic index from three independent experiments. Error bars indicate SEM. **(F)** HeLa cells expressing MPS1 GFP-MPS1^WT^, MPS1^S281A^, or MPS1^S281D^ were imaged every 2 min as they passed through mitosis. GFP-MPS1 and DNA visualized using SiR-Hoechst are shown. Arrows mark DNA bridges. **(G)** NEBD-anaphase duration is plotted; each point represents an individual cell with mean NEBD-anaphase time and SD shown. ****, P < 0.0001 (Student’s *t* test). **(H)** Cumulative mitotic exit frequency of cells expressing either GFP-MPS1^WT^ (*n* = 30), GFP-MPS1^S281A^ (*n* = 28), or GFP-MPS1^S281D^ (*n* = 25). **(I)** Simulation of checkpoint regulated mitotic exit for MPS1^WT^ and the MPS1^S281A/D^ mutants, plotted as for the experimental data in H.

MPS1 is phosphorylated at a series of candidate CDK sites other than S281 (Dou et al., 2011). To assess the functional consequences of CDK phosphorylation at these sites, their roles in MPS1 localization were examined. This analysis revealed that only S281 is required for MPS1 localization to unattached kinetochores (Fig. 4 A). Subsequently, the role of S281 phosphorylation in checkpoint function was examined by replacing endogenous MPS1 with either WT, S281A, or S281D mutants (Fig. S3 A). Following checkpoint challenge for 5 min with nocodazole, MPS1^S281A^ remained cytoplasmic, and a MAD1 checkpoint signal was not generated at the unattached kinetochores (Fig. 4 B, middle). Conversely, WT MPS1 or the S281D phospho-mimetic were recruited to the unattached kinetochores, and a MAD1 checkpoint signal was observed (Fig. 4 B, right). Similar results were obtained using the microtubule stabilizing agent Taxol to trigger spindle defects (Fig. S3 B), showing MPS1 S281 phosphorylation is a general requirement for kinetochore localization of MPS1 and generation of a MAD1 checkpoint signal. These findings are in good agreement with the location of this site within the kinetochore binding domain of MPS1 (Liu et al., 2003; Stucke et al., 2004), rather than the kinase domain (Fig. 4 A). To assess kinase activity, purified FLAG-MPS1 proteins and GFP-MPS1 isolated from the HeLa Flp-In/TREx cell lines used for the functional assays were tested for MPS1 autophosphorylation or phosphorylation of the known substrate KNL1. For both FLAG- and GFP-MPS1, the S281A mutant had a similar level of activity to the WT protein, whereas a kinase-dead (KD) control MPS1^KD^ was inactive as expected (Fig. S3, C and D).

Kinetochore tethering of WT MPS1 has previously been reported to support spindle checkpoint arrest in an MPS1 kinase activity–dependent fashion (Jelluma et al., 2010). If the crucial role of S281 phosphorylation is in kinetochore targeting rather than kinase activation, then directly tethering MPS1^S281A^ should support checkpoint function. Backing this view, direct tethering of MPS1^S281A^ to kinetochores by fusion to the kinetochore protein Mis12 rescued its ability to promote BUBR1 recruitment in an MPS1 kinase activity–dependent fashion (Fig. 4, and D, control and MPS1-i; and Fig. S3 E). Furthermore, induction of either GFP-Mis12-MPS1^WT^ or GFP-Mis12-MPS1^S281A^ resulted in cell cycle arrest (Fig. 4 E). These findings lend support to the idea that MPS1^S281A^ is catalytically active and can initiate and sustain spindle checkpoint signaling. In contrast to our results, earlier work with *Xenopus* egg extracts had indicated that phosphorylation of the equivalent site on *Xenopus* MPS1, S283, was important for MPS1 activity rather than localization (Morin et al., 2012). While we cannot fully explain this discrepancy, which may in part be due to species differences, our results confirm that human MPS1 S281, like the *Xenopus* equivalent S283, is critical for spindle checkpoint activation.

Next, we asked if MPS1 S281 phosphorylation is required for checkpoint signaling during an unperturbed cell cycle. Compared with MPS1 WT controls and MPS1^S281D^, MPS1^S281A^ cells underwent significantly shortened mitosis and displayed chromosome bridges in anaphase (Fig. 4, F–H). Importantly, no significant differences in the length of mitosis, from NEBD to anaphase onset, between MPS1 WT and MPS1^S281D^ cells were observed (Fig. 4, F–H). This is consistent with the notion that CDK phosphorylation of MPS1 plays a role in enabling the checkpoint response in mitosis and does not contribute to the dynamics of checkpoint signaling during error correction. Remarkably, population-level simulations of MPS1 behavior using our updated model for the spindle checkpoint (Fig. 3 A) recapitulated the accelerated mitotic progression seen in MPS1^S281A^-expressing cells (Fig. 4 I) and was a close fit for experimental data (compare Fig. 4, H and I).

These findings agree with the proposal that CDK1-CCNB1 and PP2A-B55 activities converge on a common target, MPS1. By regulating the level of MPS1 phosphorylation at S281, they control its ability to bind to unattached kinetochores and promote checkpoint signaling. MPS1 will remain phosphorylated and retain the ability to respond to unattached kinetochores after initial checkpoint silencing, because PP2A-B55 activation is delayed due to slow decay in the levels of its phosphorylated inhibitor, pENSA. This property of the B55-regulatory system is most clearly seen by temporal simulation of the experiments shown in Fig. 1 (B and D). Following CDK inhibition, total MPS1 phosphorylation (pMPS1) drops more slowly than CDK1 activity, since PP2A-B55 remains inhibited by phosphorylated ENSA (Fig. S4 A, right). However, a spindle defect in this time (shown as nocodazole treatment) will rapidly generate unattached kinetochores that recruit phosphorylated MPS1 (Fig. S4 A, pMPS1^KT^ +CDK-i +Noc). Thus, the checkpoint initially responds, but then inactivates, because pMPS1 continues to fall past the threshold required for checkpoint signaling. This transient recruitment of MPS1 to kinetochores was confirmed by checkpoint challenge of cells exiting mitosis (Fig. S4 C, white arrows), where CDK1-CCNB1 activity is rapidly falling. Importantly, removal of PP2A-B55 stabilizes the checkpoint system upon CDK1 inhibition so that kinetochores retain the ability to recruit pMPS1 for longer (Fig. S4 B, pMPS1^KT^ +CDK-i +Noc).

### Aurora B and CDK1 contribute to MPS1 kinetochore localization

**Figure 5.**
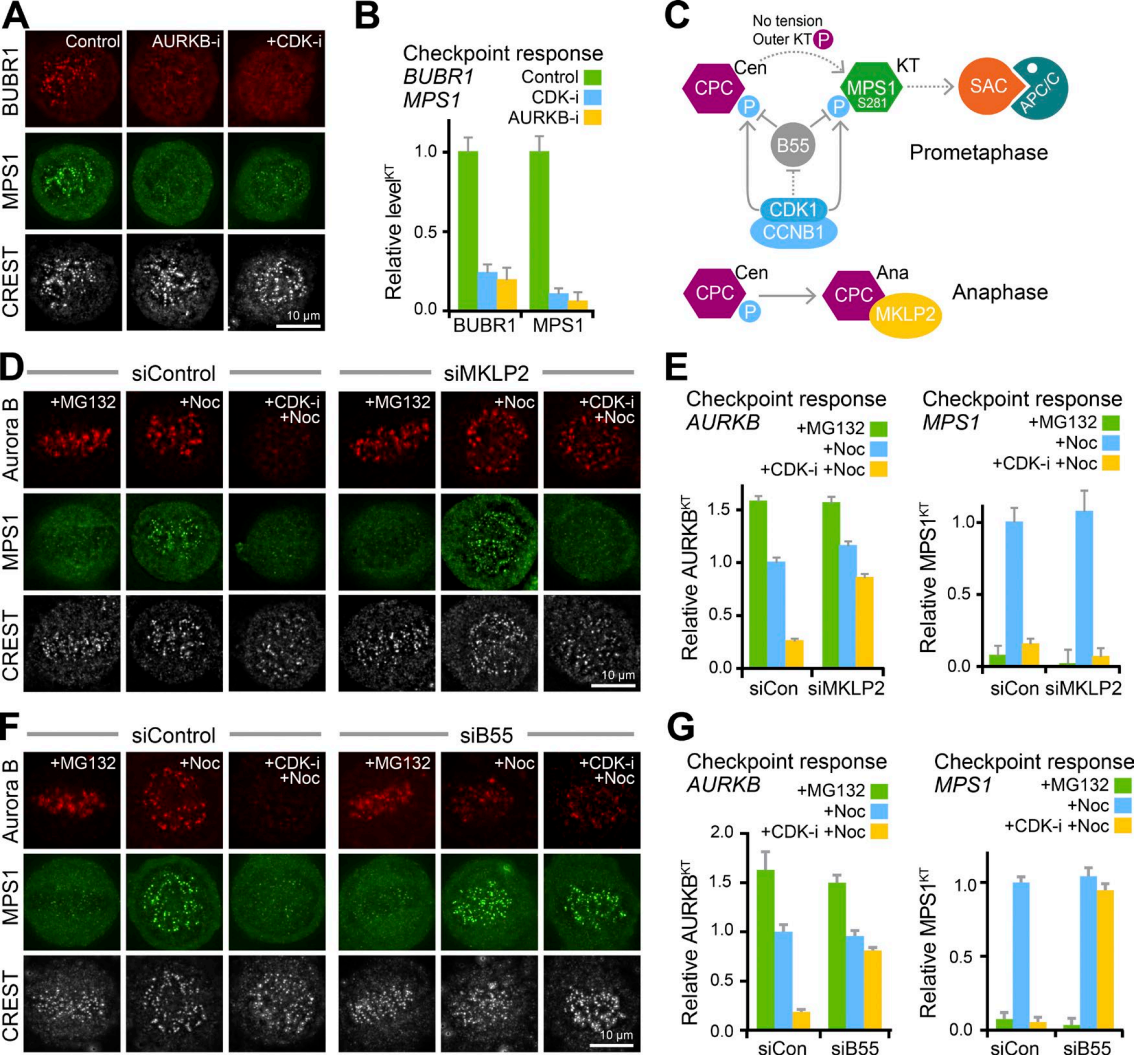
**CDK1 and Aurora B are necessary for MPS1 recruitment to unattached kinetochores. (A)** HeLa MPS1-GFP cells were arrested for 2.5 h with 20 µM MG132 and then treated with 2 µM ZM447439 (+AURKB-i) or 5 µM flavopiridol (+CDK-i) for 10 min. BUBR1 and kinetochores (CREST) were detected using antibodies, and MPS1 was detected using GFP fluorescence. **(B)** Kinetochore-associated BUBR1 and MPS1-GFP normalized to the mean of the nocodazole-treated control are plotted; error bars indicate the SEM (≥5 cells and 15 kinetochores measured per cell for three independent experiments). **(C)** Schematic showing the proposed relationship between CDK1-CCNB1 and PP2A-B55 activities and centromeric Aurora B (CPC Cen) and kinetochore MPS1 (MPS1 KT) localization. The bottom panel shows the role of MKLP2 in transport of the Aurora B CPC from centromeres to the anaphase spindle. **(D)** MPS1 and Aurora B localization were followed in control (siControl) and MKLP2-depleted (siMKLP2) HeLa MPS1-GFP cells arrested for 2.5 h with 20 µM MG132 and then either fixed immediately (+MG132), treated with 3 µM nocodazole for 5 min (+Noc) or 5 µM flavopiridol for 1 min, followed by addition of 3 µM nocodazole for 5 min (+CDK-i +Noc). Aurora B and kinetochores (CREST) were detected using antibodies, and MPS1 was detected using GFP fluorescence. Aurora B and kinetochores (CREST) were detected using antibodies, and MPS1 was detected using GFP fluorescence. **(E)** Kinetochore-associated Aurora B and MPS1-GFP normalized to the mean of the nocodazole treated control are plotted; error bars indicate the SEM (≥5 cells and 15 kinetochores measured per cell for three independent experiments). **(F)** MPS1 and Aurora B localization were followed in siControl and PP2A-B55–depleted (siB55) HeLa MPS1-GFP cells. **(G)** Kinetochore associated Aurora B and MPS1-GFP normalized to the mean of the nocodazole treated control are plotted, error bars indicate the SEM (≥5 cells and 15 kinetochores measured per cell for three independent experiments).

Our results so far show CDK1 activity is needed for localization of MPS1 to kinetochores. As discussed already, centromeric Aurora B activity is a further prerequisite for MPS1 recruitment to unattached kinetochores (Saurin et al., 2011; Nijenhuis et al., 2013; Zhu et al., 2013). We therefore examined the relationship between CDK1 and Aurora B activities and found that both are required for MPS1 localization to kinetochores and generation of a checkpoint signal (Fig. 5, A and B). Checkpoint signaling initiated by MPS1 is thus dependent on both Aurora B and CDK1 activities (Fig. 5 C). CDK1-CCNB is placed upstream of these other components in the schematic because centromeric Aurora B localization is dependent on CDK1 activity in checkpoint-active cells (Fig. 5, D and E; siControl; Tsukahara et al., 2010). To further dissect the relative importance of CDK1 and Aurora B activities for MPS1 localization, we exploited the fact that transport of Aurora B away from centromeres following a drop in CDK1 activity in anaphase requires the mitotic kinesin MKLP2 (Gruneberg et al., 2004; Hümmer and Mayer, 2009; Fig. S5, A–C). This provides a means to separate the contributions of Aurora B and CDK1 to MPS1 localization. As expected, depletion of MKLP2 in checkpoint-arrested cells resulted in retention of Aurora B at the centromere when CDK1 activity was inhibited (Fig. 5, D and E; siMKLP2). This creates a situation where Aurora B activity is present, but CDK1 activity is not. Under these conditions, MPS1 was rapidly lost from kinetochores (Fig. 5, D and E), showing that centromeric Aurora B in the absence of CDK1 activity is not sufficient to support MPS1 localization or a checkpoint response. In anaphase cells where CDK1 activity is limiting due to destruction of CCNB1, cell cycle progression was unaltered in MKLP2-depleted cells, and compared with prometaphase cells, only low levels of MPS1 were observed at kinetochores, even in the absence of MKLP2 (Fig. S5, A and D–F). CDK1 activity is therefore a limiting factor for MPS1 recruitment to kinetochores independent of its role in Aurora B regulation. To further test this idea, the effects of depleting the CDK1-counteracting phosphatase PP2A-B55 were investigated. In the absence of PP2A-B55 both centromeric Aurora B and kinetochore MPS1 were maintained when CDK activity was inhibited (Fig. 5, F and G). Together these results show that CDK1 and PP2A-B55 regulate both Aurora B and MPS1 localizations (Fig. 5 C) and hence the checkpoint response. The role of PP2A-B55 activity in limiting the checkpoint-responsive window was therefore investigated further.

### PP2A-B55 and MASTL set the CCNB1 threshold for a checkpoint-permissive state

CDK1-CCNB1 and PP2A-B55 activities are linked due to the MASTL/Greatwall-dependent generation of phosphorylated ENSA/ARPP19 (Castilho et al., 2009; Gharbi-Ayachi et al., 2010; Mochida et al., 2010). Because MASTL is activated by CDK1 phosphorylation, PP2A-B55 only becomes active once CCNB1 drops below a critical threshold (Blake-Hodek et al., 2012; Cundell et al., 2013). Depletion of MASTL uncouples this relationship and results in a constitutive maximal level of PP2A-B55 activity in mitosis, leading to precocious dephosphorylation of CDK1-targets during mitotic exit (Cundell et al., 2013). Indeed, in the case of MPS1, steady-state phosphorylation at S281 is already reduced by ∼40% in metaphase, and loss of this phosphorylation occurs more rapidly in anaphase (Fig. 3 D and Fig. S2 E). The CDK1-threshold for PP2A-B55 activation during mitotic exit creates a time window, after the initial silencing at the end of prometaphase, in which the checkpoint can still respond. This ensures that surveillance for spindle defects continues until the point of no return, when chromosome segregation is initiated. Removal of this threshold would commit cells to mitotic exit at the time of initial checkpoint silencing and thus curtail ongoing surveillance for spindle defects. We therefore set out to determine the concentration of CCNB1 required to support the checkpoint-permissive state and to dissect the role of PP2A-B55 and its regulatory pathway in setting this threshold.

**Figure 6.**
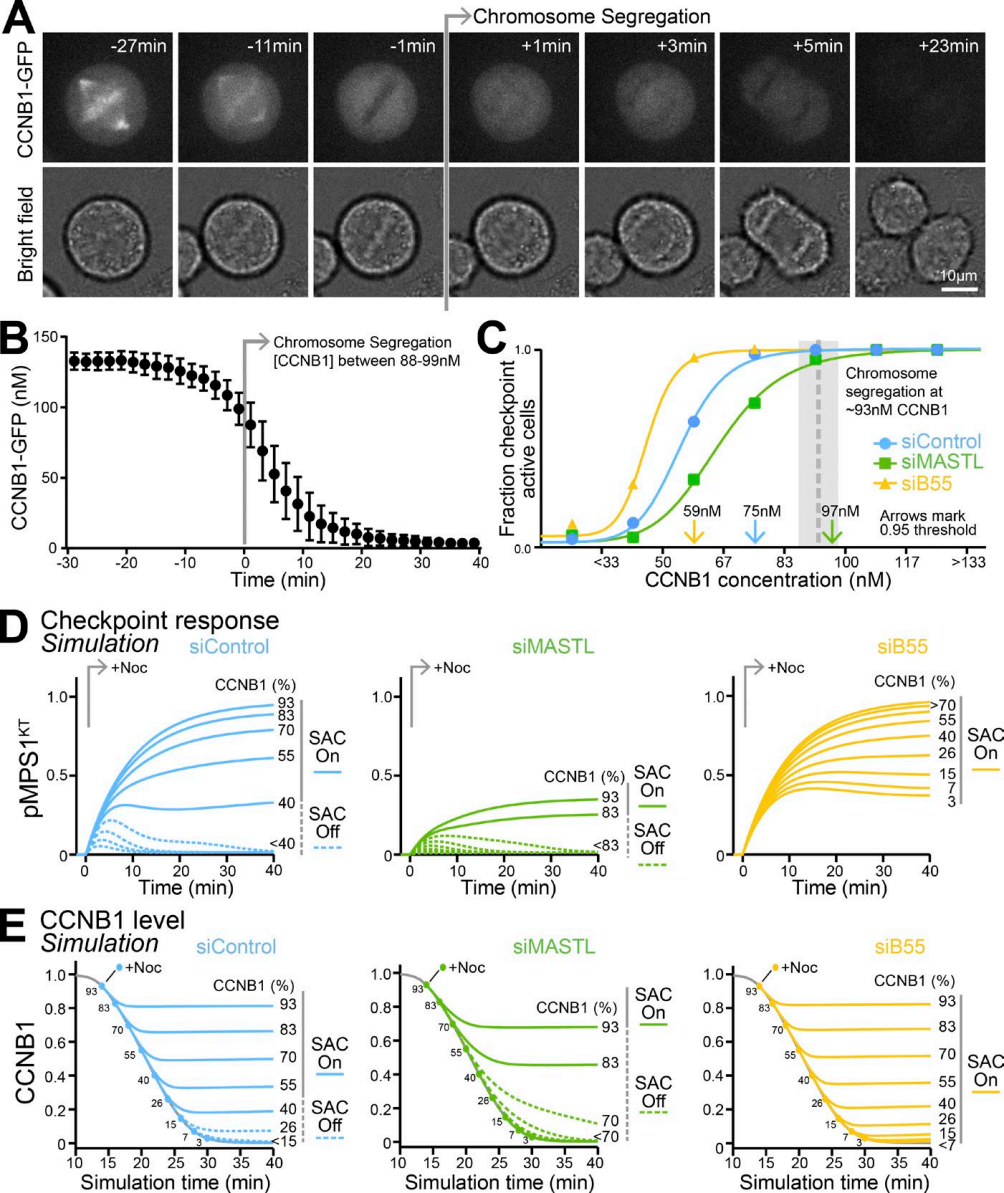
**PP2A-B55 and MASTL determine the CCNB1 threshold for checkpoint signaling. (A)** CCNB1 was measured every 2 min in HeLa CCNB1-GFP cells exiting mitosis; see images. Timings are relative to chromosome segregation. **(B)** The graph shows mean CCNB1-GFP calibrated to a steady-state level of 140 nM CCNB1 in mitosis; bars indicate the SEM (*n* = 7). **(C)** Populations of control (siControl), MASTL-depleted (siMASTL), and PP2A-B55–depleted (siB55) HeLa CCNB1-GFP cells were released from a thymidine block for 10 h and then challenged with 3 µM nocodazole for 5 min and stained for MAD1. Checkpoint status and total CCNB1 levels were determined. The fraction of checkpoint-active cells was plotted for bins of 33 nM CCNB1 on the graph for *n* = 880, 895, or 657 cells for siControl, siB55, and siMASTL, respectively. Colored arrows mark the CCNB1 concentration, below which checkpoint activity became unstable, defined as the 95% threshold. **(D)** Simulation of spindle checkpoint reactivation at different levels of CCNB1 for the siControl (MASTL = 1.0 and B55 = 1.0), siMASTL (MASTL = 0.1 and B55 = 1.0), and siB55 (MASTL = 1.0 and B55 = 0.2) conditions. Checkpoint response, measured by generation of S281 phosphorylated MPS1 at kinetochores (pMPS1^KT^), is plotted as a function of time in the line graphs. **(E)** Simulation of CCNB1 stability in unperturbed mitotic exit (gray line) and after checkpoint reactivation (+Noc) for siControl, siMASTL (MASTL = 0.1 and B55 = 1.0), and siB55 (MASTL = 1.0 and B55 = 0.2) conditions. Numbers indicate CCNB1 level at the time of Noc addition and the corresponding output curve for CCNB1. Solid lines indicate conditions where CCNB1 was stabilized, i.e., the spindle checkpoint was stably active (SAC On), and dotted lines indicate that CCNB1 was destroyed, i.e., the checkpoint failed to become stably active (SAC Off).

HeLa cells expressing GFP-tagged CCNB1 at the endogenous locus were used to measure the threshold level of CCNB1 at which chromosome segregation occurs. These measurements were calibrated against quantitative proteomics showing the maximal CCNB1 concentration is ∼140 nM in HeLa cells (Cundell et al., 2016). This approach showed that chromosome segregation is initiated at ∼100 nM CCNB1, a drop of ∼30% from the 140-nM steady-state value in mitosis (Fig. 6, A and B). These measurements match previous observations that chromosome segregation occurs after ∼35% of securin has been destroyed and that this threshold is not altered by MASTL or PP2A-B55 depletion (Cundell et al., 2013).

To understand how this threshold compared with the CCNB1 levels required for checkpoint signaling, MAD1 localization to kinetochores after 5-min exposure to nocodazole was measured as a function of CCNB1 concentration for a large population of cells passing through mitosis. Compared with the control cells (siControl), depletion of PP2A-B55 (siB55) or MASTL (siMASTL) shifted the CCNB1 threshold for checkpoint activity to lower or higher concentrations, respectively (Fig. 6 C). With this approach, the CCNB1 thresholds for spindle checkpoint signaling are estimated to be 75 nM for the control, <60 nM for PP2A-B55, and ∼100 nM for MASTL-depleted cells (Fig. 6 C). In combination, these data show that the window of checkpoint responsiveness determined by CCNB1 levels is poised so that a checkpoint response can be initiated up to the point of chromosome segregation. Depletion of MASTL shifts the level of CCNB1 required to permit checkpoint signaling close to the level for chromosome segregation, potentially curtailing a checkpoint response.

### PP2A-B55 and MASTL determine the point of no return in mitotic exit

**Figure 7.**
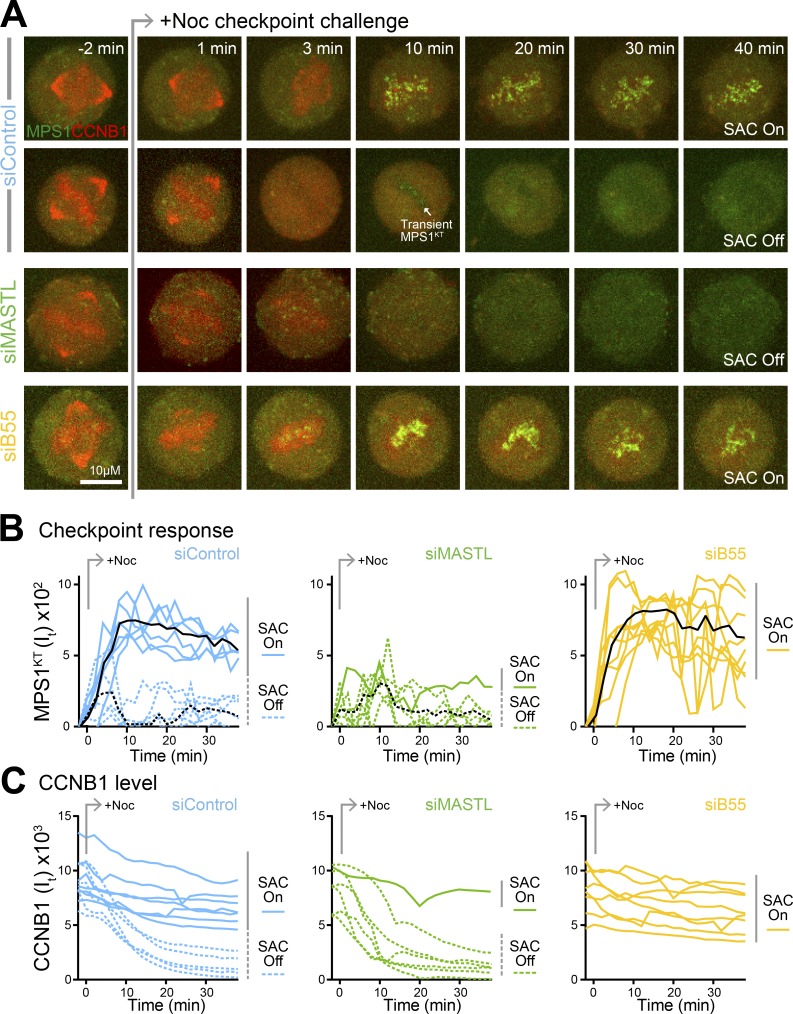
**The anaphase checkpoint reactivation threshold is set by PP2A-B55 and MASTL. (A)** Control (siControl), MASTL-depleted (siMASTL), and PP2A-B55–depleted (siB55) HeLa MPS1-GFP CCNB1-mCherry cells were imaged every 1 min until metaphase, identified by spindle architecture, defined by CCNB1 and the lack of MPS1-positive kinetochores, was reached. Checkpoint-silenced cells were then challenged with 3 µM nocodazole and imaged every 1 min for 40 min. **(B and C)** Fluorescence intensity (I_t_) for MPS1-GFP at kinetochores (B) and total cellular CCNB1-mCherry (C) are plotted over time for single cells (*n* = 13 for siControl, 7 for siMASTL, and 8 for siB55). Color-coded solid lines mark cells showing a spindle checkpoint (SAC) response, and dotted lines mark cells where the checkpoint remained silent. For MPS1, the black lines mark the mean intensity as a function of time for checkpoint active or silent cells.

To test the consequences of this regulatory mechanism, modeling of checkpoint signaling was combined with live imaging of the checkpoint response in MPS1-GFP/CCNB1-mCherry HeLa cells. Simulation of the checkpoint response at different levels of CCNB1, corresponding to cells at different stages of mitotic exit, revealed a discrete threshold for MPS1 recruitment to kinetochores (Fig. 6 D, siControl), and stable checkpoint reactivation defined by the stabilization of CCNB1 (Fig. 6 E) under control conditions. Reduction of MASTL activity resulted in stable checkpoint signaling only when CCNB1 was close to its maximal value (Fig. 6, D and E; siMASTL). In contrast, the checkpoint always showed a stable response upon reduction of PP2A-B55 (Fig. 6, D and E; siB55). For experimental tests of this model, metaphase, checkpoint-silenced cells were identified by the loss of MPS1 fluorescence from kinetochores. A challenge with 3 µM nocodazole was used to test if cells had passed beyond the CCNB1 threshold supporting the checkpoint response. In almost all instances (86%), MASTL-depleted cells failed to recruit MPS1 to kinetochores (Fig. 7, A and B; and Video 2), reactivate the checkpoint, and stabilize CCNB1 levels (Fig. 7 C). In contrast, PP2A-B55–depleted cells always (100%) displayed MPS1 recruitment to kinetochores and a robust checkpoint response leading to stabilization of CCNB1 (Fig. 7, A–C; and Video 2). This correlates with the shift in CCNB1 threshold needed to support a checkpoint-permissive state to higher or lower values in MASTL and PP2A-B55–depleted cells, respectively (Fig. 6 C). Importantly, control cells that have an intermediate CCNB1 checkpoint threshold showed both behaviors, either reactivating the checkpoint and stabilizing CCNB1 (54% of cells; Fig. 7, A–C; SAC On) or failing to sustain checkpoint activity, thus enabling CCNB1 destruction (46% of cells; Fig. 7, A–C; SAC Off). MPS1 is initially recruited to kinetochores in both cases; however, in the latter state this is only transient, and the response is curtailed by rising PP2A-B55 activity (Fig. S4).

We therefore conclude that the checkpoint response to spindle defects is dependent on the relationship between PP2A-B55 activity and the precise level of CCNB1, as defined in our model (Fig. 3 A). PP2A-B55 and its MASTL-dependent regulatory system therefore set the threshold for the checkpoint-permissive state and ensure this lies after the point of no return, when chromosomes segregate. This enables even late spindle defects to halt mitotic exit and thus prevents the generation of aneuploid cells.

## Discussion

CDK1-CCNB1 creates a spindle checkpoint–permissive state because it both promotes MPS1-dependent checkpoint signaling from unattached kinetochores and inhibits a checkpoint inactivating phosphatase (He et al., 2011). At the onset of anaphase, this response to unattached kinetochores is terminated in two ways. First, destruction of CCNB1 removes a core component of the checkpoint pathway and prevents further activating phosphorylation required for checkpoint signaling. CCNB1 also behaves as a canonical spindle checkpoint protein and localizes to unattached kinetochores in an MPS1- and MAD1-dependent fashion (see Alfonso-Pérez et al. in this issue). Second, CDK1-CCNB1 maintains MPS1 activity by inhibiting the S281-counteracting phosphatase PP2A-B55. Uncoupling the B55-regulatory pathway results in the premature loss of the checkpoint-permissive state and thus the potential to initiate checkpoint signaling during the early stages of mitotic exit. This clarifies why MASTL-depleted cells fail to maintain a checkpoint arrest (Manchado et al., 2010; Diril et al., 2016) and thus undergo precocious cytokinesis (Voets and Wolthuis, 2010; Cundell et al., 2013). Localization of Aurora B to centromeres is also controlled by this pathway, and previous work suggests that this is in part due to CDK phosphorylation of the candidate PP2A-B55 site at T59 of the INCENP subunit of the Aurora B CPC (Hümmer and Mayer, 2009; Hein et al., 2017). This explains how CDK1-CCNB and Aurora B activities combine to support MPS1 localization to unattached kinetochores. It has previously been proposed that Aurora B relocalization from the centromeres to the central spindle at the metaphase to anaphase transition is a key event limiting MPS1 recruitment to kinetochores and spindle checkpoint signaling (Vázquez-Novelle and Petronczki, 2010). However, as demonstrated here, centromeric Aurora B activity alone is insufficient for either effective MPS1 recruitment to kinetochores or spindle checkpoint signaling in prometaphase. In anaphase, when cells have degraded the bulk of CCNB1, a low level of MPS1, presumably due to a residual pool of S281-phosphorylated MPS1, can be detected at kinetochores in MKLP2-depleted cells that retain Aurora B at centromeres. However, this is a small fraction of the level seen at unattached kinetochores in prometaphase and does not generate a checkpoint response.

We therefore conclude that CDK1-CCNB1 and PP2A-B55 sit at the top of the hierarchy of kinases and phosphatases regulating checkpoint signaling and thus create a toggle switch governing the activity of downstream components, including MPS1 and Aurora B. During prometaphase, the strength of the checkpoint signal is proportionate to the amount of spindle damage, signaled by a variable number of unattached kinetochores and the amount of MAD2 accumulated at kinetochores. The spindle checkpoint thus behaves like a rheostat (Collin et al., 2013; Dick and Gerlich, 2013). We propose that this rheostat-like component of the spindle checkpoint is embedded in a toggle switch created by the action of CDK1-CCNB1 and PP2A-B55 on MPS1 and Aurora B. In this way, the analogue input created by a variable number of unattached kinetochores is converted into a digital output determining the fate of the cell. In the permissive state, the level of checkpoint signal is proportionate to the number of unattached kinetochores, in agreement with previous work (Collin et al., 2013; Dick and Gerlich, 2013).

In summary, by combining experimental data with a framework for checkpoint signaling, we explain how CDK1-CCNB1 and the B55-regulated exit pathway work together to sustain a spindle checkpoint–permissive state, responsive to spindle defects for the duration of mitosis, but not beyond. This model currently accounts for MPS1 as the primary sensor of unattached kinetochores. Furthermore, it provides a logical framework work that can be extended to other components of the spindle checkpoint and error-correction systems in future studies.

## Materials and methods

### Reagents and antibodies

General laboratory chemicals and reagents were obtained from Sigma-Aldrich and Thermo Fisher Scientific, unless specifically indicated. Inhibitors were obtained from Sigma-Aldrich (Cdk inhibitor flavopiridol, 5 mM stock), Tocris Bioscience (MPS1 inhibitor AZ3146, 20 mM stock; PP1 and PP2A inhibitors calyculin A, 1 mM stock; PP1 inhibitor tautomycetin, 2.5 mM stock; and haspin inhibitor 5-iodotubercidin, 5 mM stock), Enzo Life Sciences (microcystin-LR, 2 mM stock, and okadaic acid, 500 µM stock), Insight Bioscience (proteasome inhibitor MG132, 20 mM stock), Merck (microtubule polymerization inhibitor nocodazole, 6 mM stock), Cytoskeleton Inc. (microtubule depolymerization inhibitor Taxol, 0.1 mM stock), and Cambridge Bioscience (Eg5 inhibitor Monastrol, 100 mM stock). Inhibitor stocks were prepared in DMSO. Thymidine (Sigma-Aldrich, 100 mM stock) and doxycycline (Invivogen, 2 mM stock) were dissolved in water. DNA vital dye SiR-Hoechst (Spirochrome) was dissolved in DMSO and used at 50 nM final concentration.

Commercially available polyclonal antibodies (pAb) or monoclonal antibodies (mAb) were used for BUB1 (mouse mAb; Abcam, ab54893), MAD1 (rabbit pAb; Genetex, GTX105079), CCNB1 (mouse mAb; Millipore, [GNS3] 05-373), BUBR1 (rabbit pAb; Bethyl, A33-386A), β Actin (HRP conjugated; mouse mAb; Abcam, [AC-15] ab49900), Tubulin (mouse mAb; Sigma-Aldrich, [DM1A] T6199), PPP2R2A (mouse mAb; Cell Signaling, [2G9] 5689S), PPP1CA (rabbit pAb; Bethyl, A300-904A), PPP1CC (Goat pAb; Santa Cruz, sc6108), MASTL (rabbit pAb; Bethyl Laboratories, A302-190A, or rabbit pAb; Abcam, ab135637), HEC1 (mouse mAb [9G3.23]; GeneTex, GTX70268), Aurora B (mouse mAb; BD Transduction Laboratories, [6/AIM-1] 611082), CENP-C (guinea pig pAb; MBL, PD030), FLAG epitope tag (mouse mAb; Thermo Fisher Scientific, [FG4R], MA1-91878), GFP (rabbit pAb; Abcam, ab290), and mCherry (rabbit pAb; Abcam, ab167453). Human calcinosis, Reynaud’s phenomenon, esophageal dysmotility, sclerodactyly, and telangiectasia (CREST) serum was obtained from Antibodies Inc. (15-234-0001). Antibodies against MPS1 were raised in sheep against recombinant His-tagged MPS1 (amino acids 1–260) and affinity purified against the same recombinant protein. Sheep antibodies against mCherry and GFP have been described (Bastos and Barr, 2010). Secondary donkey antibodies against mouse, rabbit, guinea pig, or sheep and labeled with Alexa Fluor 488, Alexa Fluor 555, Alexa Fluor 647, Cy5, or HRP were purchased from Molecular Probes and Jackson ImmunoResearch Laboratories, Inc., respectively. Affinity-purified primary and HRP-coupled secondary antibodies were used at 1-µg/ml final concentration. For Western blotting, proteins were separated by SDS-PAGE and transferred to nitrocellulose using a Trans-blot Turbo system (Bio-Rad). Protein concentrations were measured by Bradford assay using Protein Assay Dye Reagent Concentrate (Bio-Rad). All Western blots were revealed using ECL (GE Healthcare).

### Molecular biology

Human MPS1, CCNB1, MAD2, and MAD1 were amplified from human testis cDNA (Marathon cDNA; Takara Bio Inc.) using Pfu polymerase (Promega). Mammalian expression constructs were made using pcDNA5/FRT/TO and pcDNA4/TO vectors (Invitrogen), modified to encode the EGFP-, mCherry-, or FLAG-reading frames. Mutagenesis was performed using the QuickChange method (Agilent Technologies). DNA primers were obtained from Invitrogen. siRNA duplexes or On-target SMARTPools were obtained from GE Healthcare. PP2A-regulatory subunit B55 was depleted using a combination of four Smartpools against each isoform (L-004824-00, L-003022-005, L-019167-00, and L-032298-00 for PPP2R2A, PPP2R2B, PPP2R2C, and PPP2R2D, respectively). MAD1 was depleted using the Smartpool L-006825-00 or the oligos 5′-CCACAGGGCAGCAGCAUGAUU-3′ and 5′-CUGCUUGGCCUGACCUGCAUU-3′ against the 3′-UTR for siRNA rescue assays. MASTL was depleted using the Smartpool L-004020-00. MPS1 was depleted using three oligos against the 3′ UTR: 5′-UUGGACUGUUAUACUCUUGAA-3′, 5′-GUGGAUAGCAAGUAUAUUCUA-3′, and 5′-CUUGAAUCCCUGUGGAAAU-3′. PP1 was depleted using a combination of four siRNA duplexes: 5′-CAUCUAUGGUUUCUAGCAUUU-3′ and 5′-GAACGACCGUGGCGUCGCUUU-3′ against PPP1CA and 5′-GCGGAGUUUGACAAUGCUU-3′ and 5′-UAGAUAAACUCAACAUCGAUU-3′ against PPP1CC.

### Cell culture procedures

HeLa cells and HEK293T were cultured in DMEM with 1% (vol/vol) GlutaMAX (Life Technologies) containing 10% (vol/vol) bovine calf serum at 37°C and 5% CO_2_. RPE1 cells were cultured in DME/F-12 (Sigma-Aldrich), supplemented with 1% (vol/vol) GlutaMAX (Invitrogen) and containing 10% (vol/vol) bovine calf serum at 37°C and 5% CO_2_. For plasmid transfection and siRNA transfection, Mirus LT1 (Mirus Bio LLC) and Oligofectamine (Invitrogen), respectively, were used. HeLa cell lines with single integrated copies of the desired transgene were created using the T-Rex doxycycline-inducible Flp-In system (Invitrogen).

HeLa cells expressing GFP-MAD2 were generated using a standard transfection protocol and selected with 1.0 µg/ml puromycin. CRISPR/Cas9-edited HeLa and hTERT-RPE cell lines with inserted GFP or mCherry tags in the C termini of the CCNB1, CCNA2, and TTK/MPS1 gene products were constructed following a published protocol with some modifications (Stewart-Ornstein and Lahav, 2016). In brief, homology recombination cassettes containing the desired knock-in DNA with flanking regions of homology of 600–750 bp to the target locus were cotransfected with a version of pSpCAS9(BB) (Addgene, 48139) containing the relevant guide RNAs and modified to removed puromycin resistance. The knock-in sequences harbor the EGFP or mCherry fluorescent proteins, preceded by a glycine-serine rich flexible linker (GS), a P2A ribosome-skipping sequence, and a resistance marker (puromycin, blasticidin, or neomycin). Antibiotic-resistant clones were selected, and successful modification was confirmed by blotting.

### Proteomic analysis of checkpoint regulated mitotic exit

For each condition, 15 × 15-cm dishes were seeded at a density of 6 × 10^5^ cells in 20 ml growth medium, left to adhere for 24 h, and then transfected with siRNA duplexes targeting luciferase (control), PPPR2A-D (B55-regulatory subunits), or MASTL for 72 h. Following an additional 20 h treatment with 0.3 µM nocodazole, mitotic cells were washed three times with 50 ml prewarmed 37°C PBS, once in 50 ml 37°C growth medium, and then resuspended in 20 ml 37°C growth medium, preequilibrated to 5% CO_2_. An initial 600 µl starting sample (t = −25 min) was taken and added to 800 µl ice-cold PBS; the cells were harvested by centrifugation at 600 *g* for 30 s and then snap frozen in liquid nitrogen for storage at −80°C. This represents cells before reformation of the mitotic spindle. The remaining cell solution was incubated at 37°C and 5% CO_2_ for 25 min, until bipolar mitotic spindle formation and chromosome capture had occurred. The MPS1 inhibitor AZ3146 was then added to this cell solution to a concentration of 51.6 µM, and a 7.8-ml sample was immediately taken (t = 0 min). This is the reference sample against which all other conditions are compared. Further 600-µl samples were taken from 10 to 45 min, as indicated in the figures.

Cell pellet fractions were removed from −80°C and resuspended in 300 µl 50 mM ammonium bicarbonate, 8 M urea buffer supplemented with 200 nM microcystin-LR, and 1:100 dilution of phosphatase inhibitor cocktail on ice. Samples were vortexed vigorously to shear genomic DNA and incubated on ice for 30 min. After 30 min, the lysate was clarified by centrifugation at 20,000 *g* for 20 min at 4°C, and the supernatant was collected. A 2.5-µl sample was used to determine protein concentration using the Bradford Assay, and a 40-µl sample was taken for Western blot analysis. Proteins in the remaining supernatant were reduced using 4 mM DTT (Fluka) for 25 min at 56°C, followed by alkylation using 8 mM iodoacetamide incubation in the dark for 30 min. Excess iodoacetamide was quenched by addition of DTT to a final concentration of 8 mM. Proteins were digested first with lysyl endopeptidase (Wako Chemicals) for 4 h at 37°C in 8 M urea and then, following dilution to 2 M urea with 50 mM ammonium bicarbonate, with trypsin (Trypsin Gold; Promega) for 12 h at 37°C. Digestions were quenched by acidification to 5% (vol/vol) formic acid.

### Dimethyl labeling and titanium dioxide phosphopeptide enrichment

Tryptic peptides, equivalent to 300 µg of total supernatant protein, were bound to SepPak reverse phase C18 columns (Waters) and subjected to on-column dimethyl labeling as previously described (Boersema et al., 2009). Peptides from all time points of the dephosphorylation assay were labeled with cyanoborohydride and deuterated formaldehyde to generate a mass increase of 32 D per primary amine, referred to as the heavy label. Additionally, one aliquot of the 0-min time point was labeled with formaldehyde and cyanoborohydride to generate a mass increase of 28 D per primary amine, referred to as the light label. At this stage, 2 µg of light- and heavy-labeled peptides were analyzed to determine both the labeling efficiency (>99%) and the total proteome. Following this, 205 µg of light and heavy labeled peptides were mixed and then subjected to titanium dioxide enrichment.

Phosphopeptide enrichment was performed using micro-spin columns, packed with titanium dioxide (TopTip; Glygen). All spin steps were performed at 550 rpm, equivalent to 34 *g*, for 5 min at room temperature. Columns were washed with 65 µl elution buffer (5% ammonia solution in water) and then three times with 65 µl loading buffer (5% [vol/vol] trifluoroacetic acid, 1 M glycolic acid, and 80% [vol/vol] acetonitrile). An equal volume of loading buffer was added to the dimethyl-labeled peptide mixtures, and then phosphopeptides were bound 65 µl at a time. After binding, columns were washed once each with loading buffer, then with 0.2% (vol/vol) trifluoroacetic acid in 80% (vol/vol) acetonitrile, followed by 20% (vol/vol) acetonitrile. Isotopically coded phosphopeptides were eluted with three washes of 20 µl elution buffer into 20 µl of 10% (vol/vol) formic acid and 10% (vol/vol) DMSO.

### Online nano–liquid chromatography and tandem mass spectrometry

LC was performed using an EASY-nano-LC 1000 system (Proxeon) in which phosphopeptides were initially trapped on a 75-µm internal diameter guard column, packed with Reprosil-Gold 120 C18, 3-µm, 120-Å pores (Dr. Maisch GmbH) in solvent A (0.1% [vol/vol] formic acid in water), using a constant pressure of 500 bar. Peptides were then separated on a 45°C heated EASY-Spray column (50-cm × 75-µm internal diameter; PepMap RSLC C18; 2 µm; Thermo Fisher Scientific) using a 3-h linear 8–30% (vol/vol) acetonitrile gradient and constant 200-nl/min flow rate. For the label check and total proteome, a 2-h linear gradient was used. Peptides were introduced via an EASY-Spray nano-electrospray ion source into an Orbitrap Elite mass spectrometer (Thermo Fisher Scientific). Spectra were acquired with resolution, 30,000; m/z range, 350–1500; AGC, target 10^6^; and maximum injection time, 250 ms. The 20 most abundant peaks were fragmented using CID (AGC target 5 × 10^3^; maximum injection time, 100 ms) or ETD (AGC cation and anion target 5 × 10^3^ and 2 × 10^5^, respectively; maximum injection time, 100 ms; normalized collision energy, 35%) in a data-dependent decision tree method. Peptide identification, proteome assembly, and quantitation of heavy-to-light phosphopeptide ratios was then performed using MaxQuant (Cox et al., 2011).

### Protein expression and purification

FLAG-tagged MPS1 WT, KD, and S281A variants were expressed and purified from HEK293T cells. For each construct, two 15-cm dishes of cells were transfected with 8 µg DNA, each, for 36 h, including 12 h nocodazole arrest. Cell pellets were lysed in 1 ml lysis buffer (20 mM Tris-HCl, pH 7.4, 300 mM NaCl, 1% [vol/vol] Triton X-100, and protease inhibitor cocktail [Sigma-Aldrich]). MPS1 was immunoprecipitated from the clarified supernatants using 100 µl FLAG-agarose beads (Sigma-Aldrich). Immunoprecipitates were washed twice with lysis buffer, four times with 20 mM Tris-HCl, pH 7.4, 300 mM NaCl, 0.1% (vol/vol) Triton X-100, two times with 20 mM Tris-HCl, pH 7.4, 300 mM NaCl, and once with 100 mM Tris-HCl, pH 7.4. For the analysis of GFP-MPS1 in GFP-MPS1 HeLa Flp-In/TREx cells, 3 × 15–cm dishes at a density of 5 × 10^6^ cells, each, of cells expressing WT, KD, or S281A GFP-MPS1 were induced with doxycycline to express the respective transgenes for 36 h. Nocodazole was added for the final 12 h of induction. Cells were harvested by mitotic shake-off. Cell pellets were lysed in 1 ml lysis buffer (20 mM Tris-HCl, pH 7.4, 300 mM NaCl, 1% [vol/vol] Triton X-100, and protease inhibitor cocktail [Sigma-Aldrich]). GFP-MPS1 was immunoprecipitated from the clarified supernatants using protein A–Dynabeads (Invitrogen) and 4 µg rabbit anti-GFP. Immunoprecipitates were washed twice with lysis buffer, four times with 20 mM Tris-HCl, pH 7.4, 300 mM NaCl, and 0.1% (vol/vol) Triton X-100; two times with 20 mM Tris-HCl, pH 7.4, and 300 mM NaCl; and once with 100 mM Tris-HCl, pH 7.4. GST-tagged KNL1^728–1,200^ was expressed and purified as described before (Espert et al., 2014).

### MPS1 kinase assays

For kinase assays, 1 µg recombinant GST-Knl1^728-1200^ was phosphorylated with 1 µg recombinant FLAG-Mps1 WT/KD/S281A on FLAG-agarose beads or 50 ng GFP-MPS1 WT/KD/S281A on protein A-Dynabeads for 30 min at 30°C in 50 mM Tris-HCl, pH 7.3, 50 mM KCl, 10 mM MgCl_2_, 20 mM sodium β-glycerophosphate, 15 mM EGTA, 0.1 mM ATP, 1 mM DTT, and 1 µCi [^32^P]γ-ATP per reaction. Incorporation of γ^32^P into GST-KNL1^728–1,200^ and MPS1 was used as a readout of kinase activity.

### MPS1 dephosphorylation assay

Mitotically phosphorylated MPS1-GFP was isolated from 20 mg of nocodazole-arrested HeLa MPS1-GFP cell lysate and incubated with 50 nM PP2A-B55 or a buffer control in reaction buffer (20 mM Tris-HCL, pH 7.5, 150 mM NaCl, 0.1% [vol/vol] IGEPAL, 0.2 mg/ml BSA, 1 mM DTT, and 1 mM MnCl_2_) in BSA-blocked Eppendorf tubes for 30 min at 37°C (Cundell et al., 2013). The reactions were stopped by adding 100 µl urea buffer (50 mM Tris, pH 8.5, and 8 M urea) and incubating for 30 min at room temperature. The samples were loaded onto a Vivacon 10,000 D molecular weight cut-off centrifugal concentrators (Sartorius), reduced with 4 mM DTT for 30 min at room temperature, and alkylated with 8 mM chloroacetamide in the dark for 30 min at room temperature. Proteins were digested with 1.5 µg trypsin in 1 M urea for subsequent analysis by mass spectrometry.

### Immunofluorescence microscopy and image processing

Cells were fixed with either PTEMF (20 mM Pipes-KOH, pH 6.8, 0.2% [vol/vol] Triton X-100, 1 mM MgCl_2_, 10 mM EGTA, and 4% [wt/vol] formaldehyde) or 3% (wt/vol) paraformaldehyde in PBS, followed by quenching with 50 mM NH_4_Cl in PBS and a 5-min cell permeabilization with 0.2% (vol/vol) Triton X-100 in PBS. Antibody dilutions were performed in PBS with 3% (wt/vol) BSA, except for anti-CDK1 antibodies that were diluted in PBS, 3% (wt/vol) BSA, and 0.2% (vol/vol) Triton X-100. Samples seeded on no. 1 thickness coverslips were imaged on a DeltaVision Core light microscopy system (GE Healthcare) using either a 60×/1.35 NA or 100×/1.4 NA objective fitted to an Olympus IX-71 microscope stand. Standard filter sets for DAPI (excitation 390/18; emission 435/48), FITC (ex. 475/28; em. 525/48), TRITC (ex. 542/27; em. 597/45), and Cy-5 (ex. 632/22; em. 676/34) were used to sequentially excite and collect fluorescence images on a CoolSnap HQ2 charge-coupled device (CCD) camera (Photometrics) using the software package softWoRx (GE Healthcare). Cells were imaged using a 0.2-µm interval and a total stack of 2 µm and deconvolved for presentation using softWoRx. Image stacks were imported into FIJI (Schindelin et al., 2012) for maximum intensity projection and saved as 8-bit TIFF files. TIFF files were imported into Illustrator CS6 (Adobe) for figure production. For quantification, imaging was performed using either a 60×/1.35 NA oil immersion objective or a 40×/0.75 NA air objective on a BX61 Olympus microscope equipped with filter sets for DAPI, EGFP/Alexa Fluor 488, Alexa Fluor 555, and Alexa Fluor 647 (Chroma Technology Corp.), a CoolSNAP HQ2 camera (Roper Scientific), and MetaMorph 7.5 imaging software (GE Healthcare). Image analysis was performed in FIJI, using images before deconvolution. Background-corrected kinetochore intensities (≥12 cells; 15 kinetochores per cell) were determined by placing a 15-pixel circular region of interest over individual kinetochores, measuring the mean pixel fluorescence, and normalizing to mean pixel intensity of the CREST channel within the same region of interest (ROI). For analysis of the CCNB1 threshold for the checkpoint-permissive state, a 40×/1.4 NA objective was used, and a single stack was captured. CCNB1 levels was measured in mitotic cells (counted as those with condensed chromosomes). Early prophase cells were excluded. A circular ROI was drawn around cells of interest. In the case of anaphase B cells, ROI was drawn over one half of the dividing cell. Subsequent analysis of kinetochore intensities and CCNB1 levels was performed in Excel (Microsoft). All immunofluorescence experiments shown are representatives of at least three independent experiments. The kinetochore intensities plotted in graphs are averages of the three independent replicates, normalized to the control condition. Error bars represent the SEM of the three independent replicates. Production of graphs was performed on Prism (GraphPad Software, Inc.) using data exported from Excel. Statistical analysis of kinetochore intensities was performed in Excel or Prism.

### Live cell microscopy and statistical analysis

Time-lapse imaging of cells with a paired control sample was performed on a DeltaVision Core light microscopy system, as described for fixed cell samples. Fluorescence images were collected on a 512 × 512–pixel EMCCD camera (QuantEM; Photometrics) using the software package softWoRx (GE Healthcare). Cells were placed in a 37°C and 5% CO_2_ environmental chamber (Tokai Hit) on the microscope stage with lens-heating collar. Cells were seeded on two-chambered, glass-bottomed dishes (Lab-Tek) at 30,000 per well. SiR-Hoechst (Spirochrome) was added 8 h before imaging at a final concentration of 100 nM. Typically, seven planes were captured per cell, 2 µm apart every 2 min, with laser powers at 2% and 25 ms exposures. Deconvolution and maximum intensity projections were performed using softWoRx, with image cropping performed using FIJI.

All other time-lapse imaging was performed using an Ultraview Vox spinning disc confocal system (Perkin Elmer) mounted on an Olympus IX81 inverted microscope, a 512- × 512-pixel EMCCD camera (ImagEM C9100-13; Hamamatsu Photonics) and Volocity software. Cells were placed in a 37°C and 5% CO_2_ environmental chamber (Tokai Hit) on the microscope stage with lens-heating collar. Imaging was performed using a 60×/1.4 NA oil immersion objective, 4–12% laser power and 30–200-ms exposure time. Typically, 19 planes, 0.6 µm apart, were imaged every 2 min. Maximum intensity projection or summed projection of the fluorescent channels was performed in FIJI. Statistical analysis of live cell imaging data (Fig. 2 C and Fig. 4 D) was performed in GraphPad Prism.

### Spindle checkpoint response and mitotic timing assays

For checkpoint response assays, 30,000 HeLa cells expressing endogenously tagged MPS1-GFP were seeded into 6-well dishes with coverslips and transfected with control (GL2), PP2A-B55, or PP1 siRNA duplexes for a total of 72 h. After thymidine block and release for 8.5 h, cells were treated for 2 h with 20 µM MG132. At 5 min before fixation, warm growth medium with nocodazole was added to obtain a final concentration of 3 µM. If CDK1 was inhibited, 5 µM flavopiridol was added 1 min before nocodazole treatment, with DMSO of the same volume used as a control. Cells were fixed in PTEMF and stained with the indicated antibodies. Human CREST antiserum was used for kinetochore staining. To assay the role of CDK1 in spindle checkpoint maintenance, GFP-MAD2–expressing HeLa cells were seeded on 35-mm glass-bottomed dishes, arrested with 0.3 µM nocodazole for 4 h; then, MG132 was added 30 min before imaging. At 1 min before imaging, 25 nM calyculin was added, imaging was initiated, and 5 µM flavopiridol was added 2 min later (t = 0 min). Chromatin was visualized with SiR-Hoechst (Spirochrome).

For checkpoint reactivation assays, HeLa cells or HeLa CCNB1-GFP MPS1-mCherry were transfected with control (GL2) and PP2A-B55 siRNA duplexes, as for checkpoint response assays, and then thymidine was synchronized. At 8 h after thymidine release, cells were arrested for 3 h with 100 µM monastrol. Monastrol was removed with three washes in 37°C PBS, followed by three washes with 37°C growth medium. Spindle reformation was allowed to progress for 20, 30, or 40 min, followed by 5-min treatment with 3 µM nocodazole before fixation. Total treatments were therefore 25, 35, or 45 min from monastrol release, equivalent to +0, +10, and +20 after bipolar spindle formation, observed to take 25 min under these conditions.

For analysis of the CCNB1 threshold for the checkpoint-permissive state, HeLa CCNB1-GFP cells were siRNA treated for 36 h, thymidine arrested for 15 h, released for 7 h, and then thymidine arrested again for 15 h. Following the second thymidine release, cells were treated with 3 µM nocodazole for 10 min before fixation at 10.5 h. For live analysis of the checkpoint-permissive state, HeLa cells expressing endogenously tagged MPS1-GFP and CCNB1-mCherry were seeded on 35-mm-diameter glass-bottomed dishes and transfected with control (GL2), PP2A-B55, or MASTL siRNA duplexes. Per dish, roughly 10 metaphase or late prophase cells were identified and imaged, with nocodazole addition at 4 min of imaging. Only those cells in metaphase (no MPS1/CCNB1 puncta at the metaphase plate before nocodazole addition) were later analyzed.

Analysis of NEBD to anaphase timing was performed by visual inspection of deconvolved chromosomes stained with SiR-Hoechst. MPS1-GFP at kinetochores was measured in the region defined by chromatin with a paired background measurement from a cytoplasmic region within the same cell. CCNB1-mCherry levels were determined for the entire cell, with a paired background measurement for correction from outside the cell.

### Mathematical modeling

Our mathematical model is an updated version of the spindle checkpoint model of He et al. (2011), supplemented with the Greatwall/MASTL-ENSA pathway (Vinod and Novak, 2015). The unified model consists of an MPS1 and an APC/C-regulatory module (Fig. 3 A). The phosphorylation state and kinetochore localization of MPS1 has been found to be regulated by CDK1 and its counteracting phosphatase, PP2A-B55, whose activity is controlled by the CDK1-dependent MASTL-ENSA pathway. APC/C activity is regulated by its diffusible, inhibitory substrate, MCC, which binds and inhibits APC/C-dependent degradation of CCNB1 and is itself inactivated by APC/C activity. The two modules are coupled into a mutually inhibitory relationship. The APC/C module has a constitutive inhibitory effect on the input of the MPS1 module (CDK1) through APC/C-dependent CCNB1 (CycB) degradation. In return, the output of the MPS1 module (Mps1p) inhibits APC/C through activation of MCC assembly, but the strength of this inhibition is determined by the fraction of unattached kinetochores (uKT; 0 ≤ uKT ≤ 1) in the cell. The antagonism between the two modules creates two mutually exclusive states: checkpoint arrest (Mps1p, CycB high and PP2A-B55, APC/C inactive) and mitotic exit (inverse conditions).

We describe the dynamics of our model in terms of ordinary differential equations (ODEs) using exclusively mass-action kinetics. The rate equations come with several kinetic constants whose values were estimated by fitting to mitotic exit experiments with three different conditions (Fig. 3, B and C). The rate constants are denoted by an initial letter “k” and are named according to the following convention: the first one or two letters after the “k” indicate the reaction (synthesis [s], degradation [d], activation [a], inactivation [i], association [as], and dissociation [di]); the remaining letters refer to the molecule that is being synthesized or degraded. All of the rate constants have a dimension of min^−1^ because the dynamic variables representing molecule concentrations are expressed on a relative scale in the model.

The level of CycB-Cdk1 and APC/C^Cdc20^ complexes is limited by their regulatory subunits CycB (Morgan, 2007) and CDC20 (Herzog et al., 2009), respectively, so these names are used in the model to denote the entire complex. The synthesis of CycB is CDK1 dependent, while CDC20 synthesis is constant. Both CycB and CDC20 are degraded in an APC/C- and proteasome (Psome)-dependent manner. MASTL (Mastl) and ENSA are phosphorylated by Cdk1 and MASTL, respectively, and dephosphorylated by both PP2A-B55–dependent and –independent reactions. Formation of active (closed) MAD2 (Mad2a) is catalyzed by pMPS1-bound unattached kinetochores (KTMps1p). Mad2a associates with free CDC20 (Cdc20f) into MCC. Free CDC20 undergoes CDK1-dependent inhibitory phosphorylation (Labit et al., 2012), and only the dephosphorylated form can bind and activate APC/C. Free MCC (containing CDC20) binds to APC/C^Cdc20^, so their complex (labeled as MCCAPC) contains two molecules of CDC20 (Izawa and Pines, 2015; Alfieri et al., 2016; Yamaguchi et al., 2016). APC/C^Cdc20^ promotes the disassembly of MCC by proteasome-dependent degradation of its CDC20 subunit. This MCC disassembly is accompanied by inactivation of MAD2.

The model can be simulated by running the “.ode” file (see Data S1) with the freely available software package XPPAUT (http://www.math.pitt.edu/~bard/xpp/xpp.html; Ermentrout, 2002). 

The parameters listed above are the values for WT cells under control conditions (siControl). To simulate siB55 and siMASTL depletions, B55T and MastlT were set to 0.2 and 0.1, respectively. To simulate S281A and S281D mutants, k_amps1_ and k_imps1_ parameters were set to 0, respectively. Simulations were run with the conditions used for experiments in the respective figure. These were as follows.

For Fig. 3 (B–D), the initial condition of uKT for prometaphase arrested cells by nocodazole was set to 1. Spindle formation after nocodazole wash-out was simulated by setting k_iuKT_ to 0.125, 0.13, and 0.06 min^−1^ in siControl, siB55, and siMASTL cells, respectively. After 25 min, the k_amad_ parameter was set to 0 to mimic the addition of MPS1 inhibitor. Simulations are plotted from this point onward.

For Fig. S4 (A and B), the initial condition of these experiments corresponds to a metaphase block by MG132 proteasome inhibitor (Psome = 0, uKT = 0, and CycB = 1). CDK1 inhibition by flavopiridol and microtubule depolymerization by nocodazole was simulated by setting the parameters of flavo to 100 and k_auKT_ to 0.1 at 4 and 5 min, respectively.

For Fig. 4 I, a population-level simulation was performed by randomly varying both the k_iuKT_ parameter and the CycB initial condition across a series of 100 individual deterministic simulations to simulate variation within the mitotic cell population. The k_iuKT_ parameter and the CycB initial conditions were sampled from lognormal distributions with means of 0.25 and 1.09, respectively. Both log standard deviations were set to 0.5. Initial conditions for all variables, with the exception of CycB, correspond to an early mitotic state with full checkpoint signaling (Psome = 0, uKT = 1, and k_iuKT_ = 0). At t = 0, kiuKT was set to the randomly sampled value, allowing the system to align chromosomes and exit from mitosis. For each individual simulation, the time for mitotic exit was defined as the time taken for CycB to fall below a threshold value of 0.1.

For Fig. 6 (D and E) and Fig. 3 E, normal mitotic progression was simulated with CycB initial value of 0.5. Checkpoint reactivation by nocodazole during mitotic exit was simulated by setting k_auKT_ to 0.1 min^-1^ at different time points. The levels of Mps1^KT^ and CycB were plotted from the time of Noc addition (Fig. 6 D) and from start of mitotic exit when uKT = 0 (Fig. 6 E), respectively. For Fig. 3 E, the level of Mps1^KT^ was recorded at t = 8 min after nocodazole addition.

### Online supplemental material

Fig. S1 and Video 1 show the CDK1 and phosphatase dependence of checkpoint activity explored in Fig. 1. Fig. S2 explains the mass spectrometry strategy with additional controls, and Table S1 contains the dataset and parameters used for the simulation in Fig. 3. Fig. S3 shows characterization of MPS1 kinase activity and supports Fig. 4. Additionally, MPS1 kinetochore dynamics are modeled and experimentally followed in Fig. S4. Fig. S5 provides additional data on the role of Aurora B and MKLP2 in MPS1 localization and checkpoint function. Video 2 shows the dynamics of checkpoint silencing and CCNB1 stability and supplements Fig. 7. Data S1 is a code file for the unified model of the spindle checkpoint and B55-regulated exit pathway.

## Supplementary Material

Supplemental Material (PDF)

Table S1 (Excel)

Video 1

Video 2

Dataset 1
