# Supplementary material for: CDK1-CCNB1 creates a spindle checkpoint–permissive state by enabling MPS1 kinetochore localization
Source: J Cell Biol. 2019 Jan 23;218(4):1182–99. doi: 10.1083/jcb.201808014 (PMC6446832; doi:10.1083/jcb.201808014)
Supplement: Supplemental Material (PDF) [file JCB_201808014_sm.pdf]

Supplemental material

Hayward et al., https://doi.org/10.1083/jcb.201808014

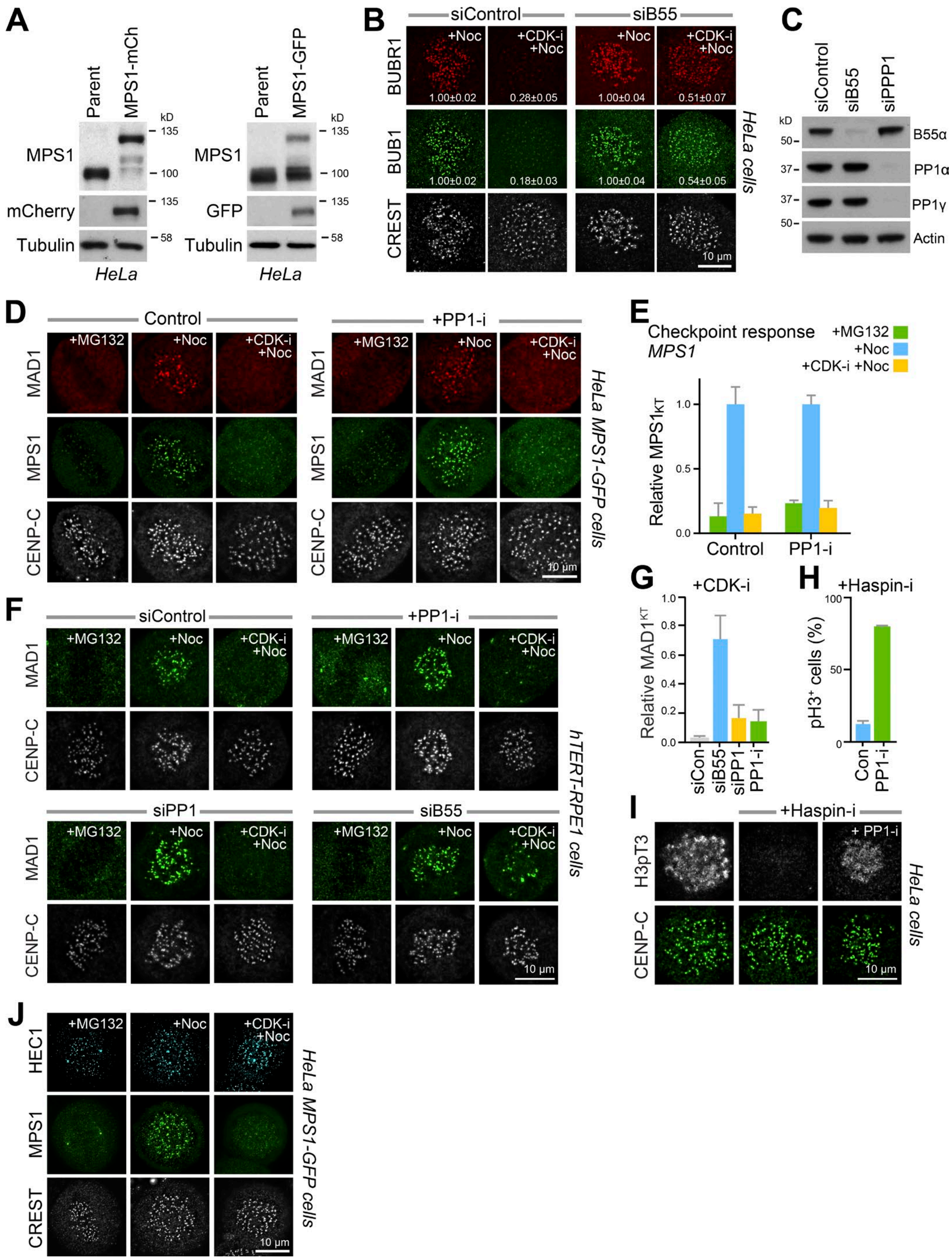

Figure S1. **Spindle checkpoint signaling is controlled by CDK1 and PP2A-B55. (A)** Western blots showing CRISPR/Cas9-mediated tagging of endogenous MPS1 with fluorescent epitope tags in HeLa cells. **(B)** Spindle checkpoint signaling was followed in control (siControl) and PP2A-B55–depleted (siB55) HeLa cells. Cells were arrested for 2.5 h with MG132 and then either fixed immediately, treated with 3 µM nocodazole for 5 min (+Noc), or with flavopiridol for 1 min, followed by addition of 3 µM nocodazole for 5 min (+CDK-i +Noc). BUB1, BUBR1, and kinetochores (CREST) were detected using antibodies. Mean levels ± SD of kinetochore-associated BUB1 and BUBR1 normalized to the mean of the nocodazole-treated control signals for the same markers are indicated as inset numbers (15 cells and 15 kinetochores measured per cell in three independent experiments). **(C)** A representative Western blot analysis of PP2A-B55 and PP1 depletion efficiencies in checkpoint silencing assays. Actin is shown as a loading control. **(D)** Checkpoint signaling and endogenous MPS1 localization were followed in HeLa cells, treated with DMSO (Control) or 5 µM tautomycetin (+PP1-i) for 25 min before nocodazole addition, as described in B. **(E)** Kinetochore-associated MPS1-GFP in control or tautomycetin-treated cells (PP1-i) was plotted; error bars indicate SEM. **(F)** hTERT-RPE-1 cells, either control depleted (siControl), treated with tautomycetin (+PP1-i), as in D, or depleted of PP1αγ (siPP1) or PP2A-B55 (siB55), were treated, as described for HeLa in B, and stained for MAD1 and CENP-C. **(G)** Mean levels of kinetochore-associated MAD1 ± SEM were plotted. **(H and I)** Nocodazole-arrested HeLa cells were either mock treated or treated with 5 µM tautomycetin for 25 min (+PP1-i) and then with 10 µM Haspin inhibitor 5-iodotubercidin (Haspin-i) for 10 min. The Haspin target histone H3 phospho-threonine 3 (H3pT3) and CENP-C were stained with antibodies. The percentage of cells showing H3pT3 staining after Haspin inhibitor treatment was plotted in H, and representative images are shown in I. **(J)** HeLa MPS1-GFP cells were treated as in B. HEC1 and kinetochores (CREST) were detected using antibodies.

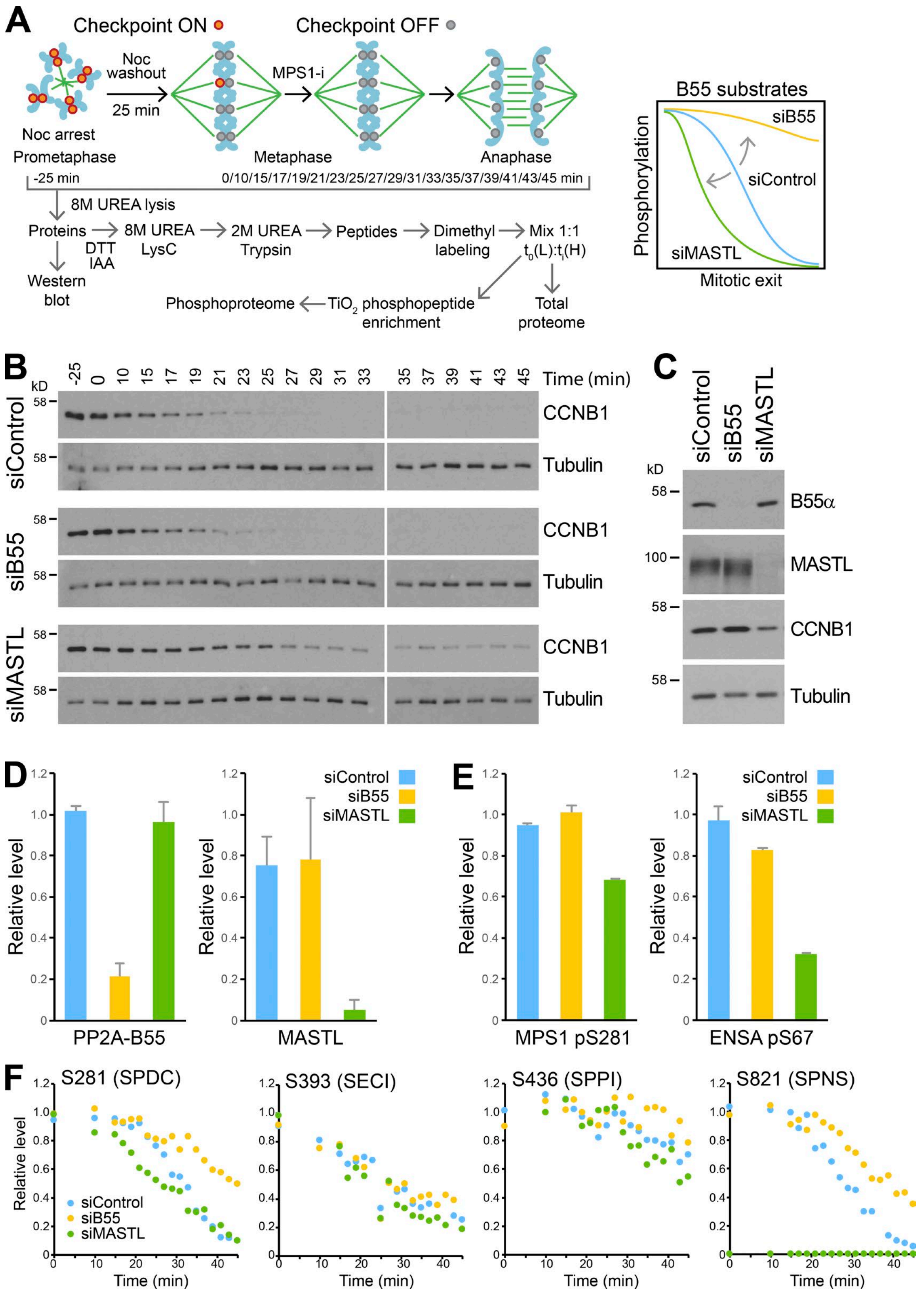

Figure S2. **Analysis of protein dephosphorylation during mitotic exit. (A)** A schematic explaining the mass spectrometry strategy used for combined analysis of protein phosphorylation status and total protein levels in cells undergoing checkpoint-regulated exit from mitosis. A cartoon of the behavior of PP2A-B55 substrates under control (siControl), PP2A-B55–depleted (siB55), and MASTL-depleted (siMASTL) conditions is shown. **(B)** Western blot analysis of CCNB1 and tubulin (loading control) levels during mitotic exit for control, siB55, and siMASTL cells. **(C)** Depletion efficiencies were confirmed by Western blotting of the PP2A-B55α–regulatory subunit or MASTL. Tubulin is a loading control. **(D)** Depletion efficiency of PP2A-B55 and MASTL calculated from mass spectrometry data are plotted; bars indicate the SEM (*n* = 2 independent experiments). **(E)** Steady-state levels of MPS1 pS281 and ENSA pS67 under siControl, siB55, and siMASTL conditions before checkpoint release were measured using mass spectrometry; bars indicate the SEM (*n* = 2). **(F)** Dephosphorylation of the indicated phospho-sites on MPS1 in siControl, siMASTL, and siB55 cells was followed by mass spectrometry.

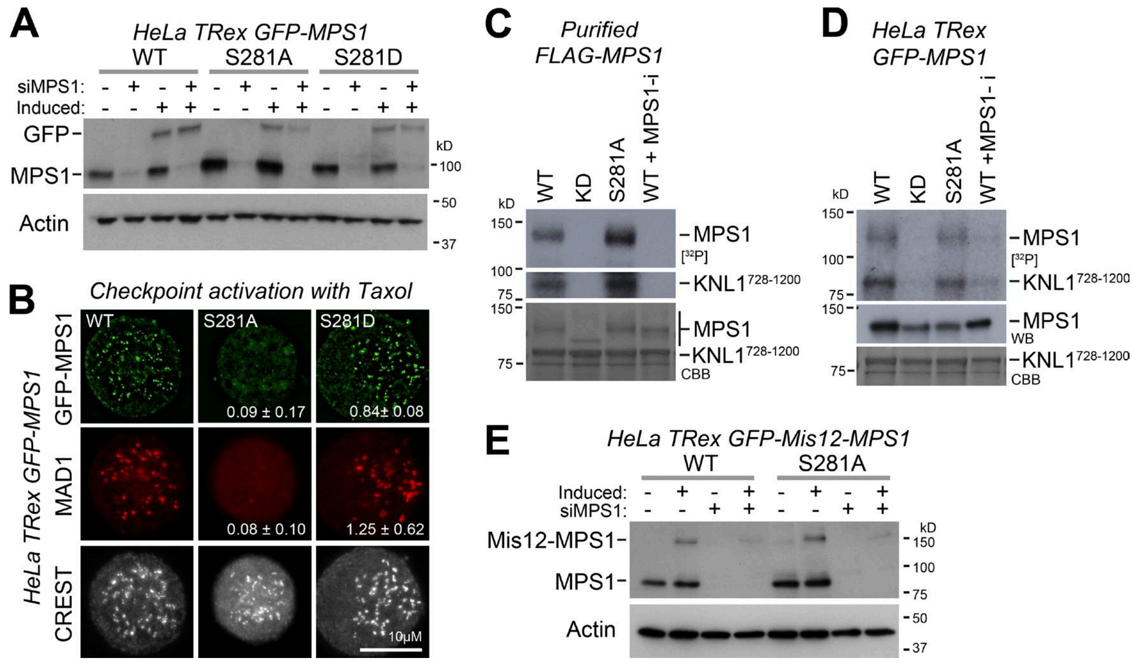

Figure S3. **MPS1 S281A fails to localize to kinetochores or activate the spindle checkpoint. (A and B)** HeLa Flp-In/TREx GFP-MPS1^{WT}, GFP-MPS1^{S281A}, or GFP-MPS1^{S281D} cells were induced for (+) or left uninduced (−) with doxycycline. Endogenous MPS1 was depleted by addition of MPS1 3′-UTR siRNA after 6 h. After 54 h, the cells were treated with 0.1 µM Taxol for 2 h. Cells were either Western blotted (A) or stained with antibodies for MAD1 and kinetochores (CREST; B). Numbers indicate mean kinetochore GFP-MPS1 or MAD1 ± SEM normalized to the mean of the MPS1^{WT} condition (*n* = 11 cells per condition with 15 kinetochores measured per cell). **(C)** Radioactive kinase assays with FLAG-MPS1^{WT}, FLAG-MPS1^{KD}, and FLAG-MPS1^{S281A}, expressed in HEK293T cells or FLAG-MPS1^{WT} + 30 µM MPS1 inhibitor (WT + MPS1-i) and GST-KNL1$^{728–1,200}$ substrate (Espert et al., 2014) were performed. Phosphorylation of GST-KNL1$^{728–1,200}$ and FLAG-MPS1 autophosphorylation was measured by ^{32}P incorporation and radiograph. Equal loading of GST KNL1$^{728–1,200}$ and FLAG-MPS1 was determined by Coomassie Brilliant Blue staining. **(D)** Radioactive kinase assays with GFP-MPS1^{WT}, GFP-MPS1^{KD}, GFP-MPS1^{S281A}, purified from HeLa Flp-In/TREx cells used in A and B, or GFP-MPS1^{WT} + 30 µM MPS1 inhibitor (WT + MPS1-i), were performed and analyzed as in C. Equal loading of GST KNL1$^{728–1,200}$ and GFP-MPS1 was determined by Coomassie Brilliant Blue staining and Western blotting with anti-MPS1 antibodies, respectively. **(E)** HeLa Flp-In/TREx GFP-Mis12-MPS1^{WT} or GFP-Mis12-MPS1^{S281A} cells were depleted of endogenous MPS1. GFP-Mis12-MPS1 transgenes were induced (+) for 24 h, and the cells were treated with 0.1 µM Taxol for 2 h. Cells were Western blotted to confirm depletion of endogenous MPS1 and induction of GFP-tagged transgenes.

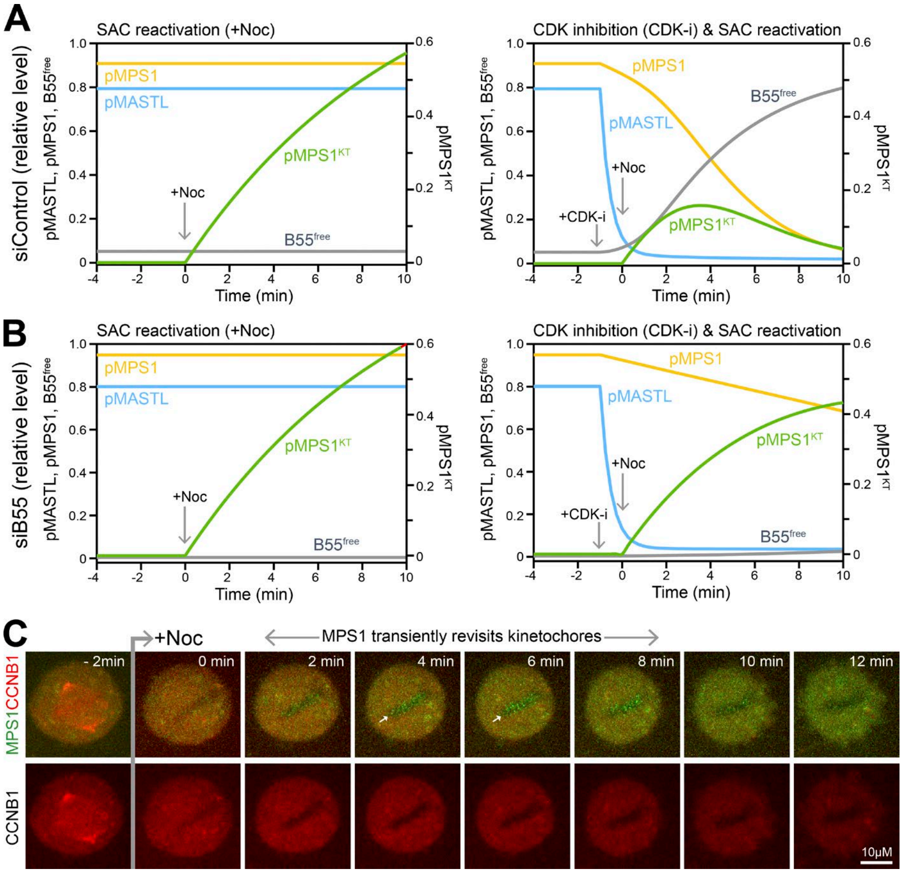

Figure S4. **Simulation explains how PP2A-B55 controls MPS1 dynamics during mitotic exit. (A and B)** Simulation of MPS1 kinetochore localization during checkpoint reactivation in control (A) and PP2A-B55–depleted (siB55; B55 = 0.2 and MASTL = 1.0) conditions (B), with or without CDK inhibition. **(C)** HeLa MPS1-GFP/CCNB1-mCherry cells in mitosis were imaged every minute until all the chromosomes had aligned and the checkpoint, visualized by MPS1, was silenced. 3 µM nocodazole was added (+Noc) to trigger checkpoint activation (defined as t = 0 min). Images from −2 min before nocodazole addition to 12 min after are shown.

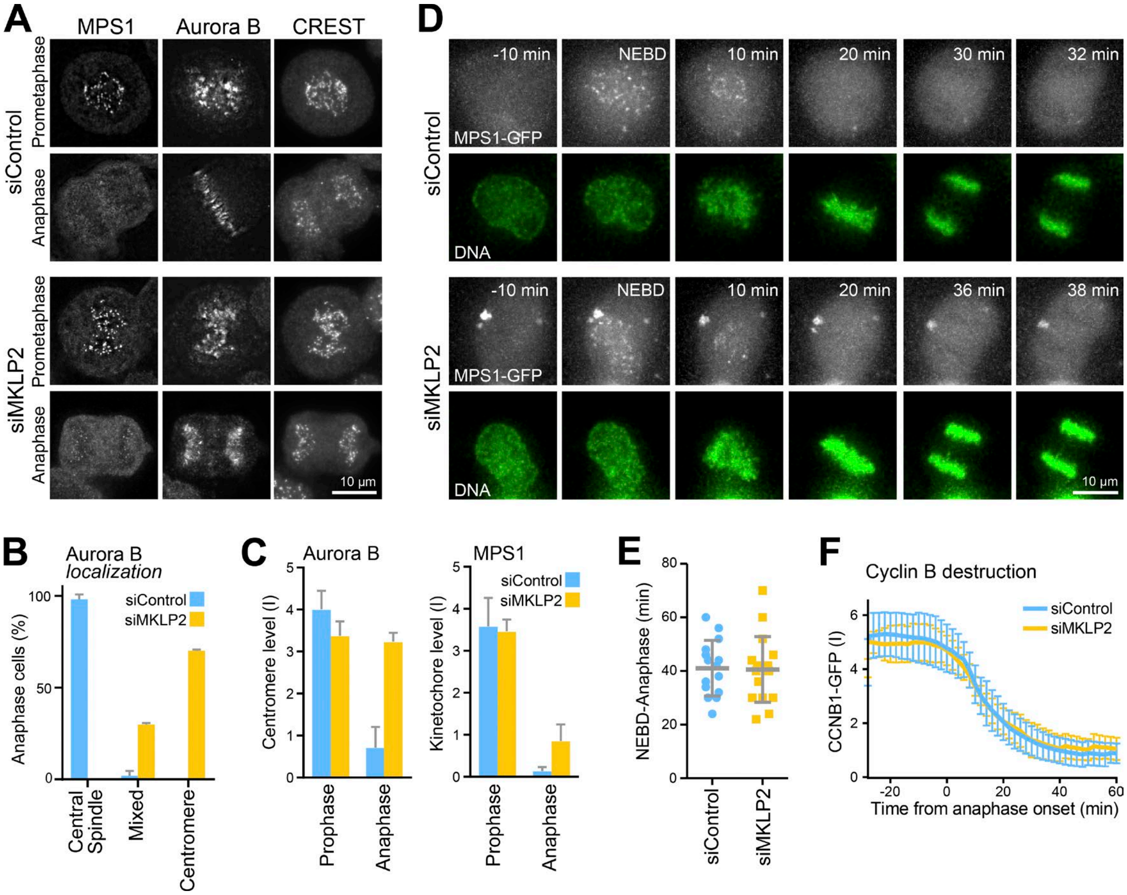

Figure S5. **MPS1 and Aurora B localization in MKLP2-depleted cells. (A)** Control (siControl) and MKLP2-depleted (siMKLP2) HeLa MPS1-GFP cells were stained for Aurora B and CREST. Examples of prometaphase and anaphase cells are shown. **(B and C)** The bar graphs show the percentage of anaphase cells with centromeric or central spindle localization of Aurora B (B) and the mean levels of Aurora B and MPS1 at centromeres and kinetochores, respectively, in both prometaphase and anaphase (C). Bars indicate the SEM (n = 2 independent experiments totaling 15 cells [siControl] and 14 cells [siMKLP2]). **(D)** siControl and siMKLP2 HeLa MPS1-GFP cells were imaged every 2 min passing through mitosis. Timings are relative to NEBD. **(E)** Scatter plot indicating the average time from NEBD to anaphase onset in control and MKLP2-depleted cells. Every dot represents one cell; the mean and the SD are shown. **(F)** The graph shows mean CCNB1-GFP intensity (arbitrary fluorescence units) in siControl and siMKLP2 HeLa CCNB1-GFP cells, imaged every 2 min exiting mitosis. Bars indicate the SEM (n = 2 independent experiments with seven cells per experiment). Timings are relative to chromosome segregation.

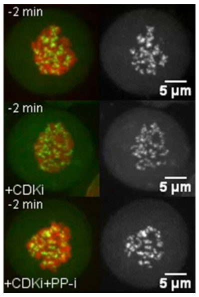

Video 1. **Related to Fig. 1.** GFP-MAD2 (green; left and grayscale)–expressing HeLa cells with addition of SiR-Hoechst at 100 nM 8 h before imaging (red) were arrested with 0.3 µM nocodaozle for 4 h and 20 µM MG132 for 15 min before imaging to prevent mitotic exit. One image stack every minute was captured, with either DMSO (−CDKi), 5 µM flavopiridol alone (+CDKi), or 25 nM calyculin A for 1 min before 5 µM flavopiridol addition (+CDKi +PP-i) at 0 min. Video is shown at 7 fps.

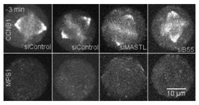

Video 2. **Related to Fig. 7**. Control (siControl), MASTL-depleted (siMASTL), and PP2A-B55–depleted (siB55) HeLa CCNB1-mCherry (top), and MPS1-GFP (bottom) cells were imaged every 2 min until metaphase, identified by spindle architecture defined by CCNB1 and lack of MPS1-positive kinetochores, was reached. These checkpoint-silenced cells were then challenged with 3 µM nocodazole before time point 1 min. Image stacks were recorded 4 min before the addition of nocodazole and subsequently every 2 min for a further 37 min. Video is shown at 7 fps.

Table S1 is included as a separate Excel file, containing the dataset and parameters used for the simulation in Fig. 3. Data S1 included as a code file for the unified model of the spindle checkpoint and B55-regulated pathway.

Reference

Espert, A., P. Uluocak, R.N. Bastos, D. Mangat, P. Graab, and U. Gruneberg. 2014. PP2A-B56 opposes Mps1 phosphorylation of Knl1 and thereby promotes spindle assembly checkpoint silencing. *J. Cell Biol.* 206:833–842. https://doi.org/10.1083/jcb.201406109
